# ChIATAC is an efficient strategy for multi-omics mapping of 3D epigenomes from low-cell inputs

**DOI:** 10.1038/s41467-023-35879-5

**Published:** 2023-01-13

**Authors:** Haoxi Chai, Harianto Tjong, Peng Li, Wei Liao, Ping Wang, Chee Hong Wong, Chew Yee Ngan, Warren J. Leonard, Chia-Lin Wei, Yijun Ruan

**Affiliations:** 1 The Jackson Laboratory for Genomic Medicine, Farmington, CT USA; 2 Laboratory of Molecular Immunology and the Immunology Center, National Heart, Lung and Blood Institute, National Institutes of Health, Bethesda, MD USA; 3 Life Sciences Institute, Zhejiang University, Hangzhou, Zhejiang Province P. R. China

**Keywords:** Genomic analysis, Chromatin structure

## Abstract

Connecting genes to their cis-regulatory elements has been enabled by genome-wide mapping of chromatin interactions using proximity ligation in ChIA-PET, Hi-C, and their derivatives. However, these methods require millions of input cells for high-quality data and thus are unsuitable for many studies when only limited cells are available. Conversely, epigenomic profiling via transposase digestion in ATAC-seq requires only hundreds to thousands of cells to robustly map open chromatin associated with transcription activity, but it cannot directly connect active genes to their distal enhancers. Here, we combine proximity ligation in ChIA-PET and transposase accessibility in ATAC-seq into ChIATAC to efficiently map interactions between open chromatin loci in low numbers of input cells. We validate ChIATAC in *Drosophila* cells and optimize it for mapping 3D epigenomes in human cells robustly. Applying ChIATAC to primary human T cells, we reveal mechanisms that topologically regulate transcriptional programs during T cell activation.

## Abstract (web summary)

Methods connecting genes to their cis-regulatory elements usually requires millions of input cells. Here the authors combine proximity ligation in ChIA-PET and transposase accessibility in ATAC-seq, reporting ChIATAC, to map interactions between open chromatin loci in low numbers of input cells.

## Introduction

The human genome comprises of 6 billion base pairs and is organized in 23 pairs of chromosomes that fold through long-range chromatin interactions into loops and domains in an ordered but also fluid manner within the confines of the nucleus^1–5^. Advanced chromatin interaction-mapping methods based on proximity ligation—such as Hi-C^6,7^ and ChIA-PET^8–10^—have greatly expanded our knowledge of 3D genome organization in human and model organisms. In particular, the inclusion of chromatin immunoprecipitation (ChIP) to specifically enrich protein factor-associated chromatin complexes in ChIA-PET and its derivatives HiChIP^11^ and PLAC-seq^12^ efficiently capture chromatin interactions associated with chromatin architectural proteins (CTCF, cohesin, etc.) and transcription factors (TF) such as RNA polymerase II (RNAPII) and other specific TFs in a genome-wide manner. However, these methods require large numbers of input cells (10^6^–10^7^) for each experiment in order to generate high-quality data, and when applied in studies with a low number of input cells, the resulting data were often of poor quality, mapping only the higher-order chromatin domains and lacking the details of individual interactions between specific chromatin loci^13,14^. Thus, they cannot be readily applied in studies of biological and clinical samples of high interest, which often have few cells available and yet require high-resolution data. Meanwhile, chromatin profiling methods such as ATAC-seq^15^, DNase-seq^16^, and FAIRE-seq^17^ map regions of open chromatin genome-wide to identify candidate promoters and *cis*-regulatory elements that are accessible to *trans*-regulatory factors. Specifically, ATAC-seq identifies chromatin that is sensitive to digestion by hyperactive Tn5 transposase, which simultaneously cuts genomic DNA and inserts a sequencing adaptor at its cutting site; this direct coupling of ‘tagmentation’ to the digestion of accessible DNA enables high-efficiency construction of DNA sequencing libraries from as few as 500 input cells^15^. However, ATAC-seq analysis cannot reveal direct interactions between open chromatin loci, and thus could not reflect accurate long-range transcriptional regulation, such as enhancer–promoter interactions.

Here, we sought to develop a method that could efficiently and simultaneously map open chromatin loci and chromatin interactions associated with transcriptional activity using low numbers of input cells. There have been several efforts to combine different chromatin-probing protocols for more efficient and informative multi-omics analysis. ChIA-PET was the first to simultaneously detect TF binding sites (TFBSs) and chromatin interactions associated with the TF of interest^8–10^. Trac-looping^18^ is another multi-omic method that uses a bivalent oligonucleotide linker, which favors the formation of a tetramer complex of transposase to tag and cut two accessible chromatin loci that are linearly faraway but spatially in proximity, thus, capturing interactions between open chromatin sites. Ocean-C^19^ and HiCAR^20^ are two of the recent methods that either includes FAIRE (Formaldehyde Assisted Isolation of Regulatory Elements) steps to separate protein-free DNA from protein-bound material or Tn5 digestion in Hi-C protocol to detect contacts between open chromatin loci. However, these methods still require large numbers (10^5^−10^6^) of input cells in order to obtain quality chromatin interaction data, thus limiting their applications.

Improving efficiency and reducing the number of input cells for broader application of chromatin interaction assays has been challenging^21^. Here, we report a strategy called ChIATAC that combines in situ proximity ligation for chromatin interaction analysis (ChIA) and the efficiency of transposase-based ATAC approach for chromatin library construction. We show that ChIATAC can simultaneously and robustly identify open chromatin loci and capture chromatin interactions from as few as 1000 input cells per library. To demonstrate the utility of ChIATAC, we used it to comprehensively characterize the 3D epigenome during T cell activation by T cell receptor (TCR) and interleukin-2 (IL-2).

## Results

### Design principle and proof of concept for ChIATAC

**Fig. 1:**
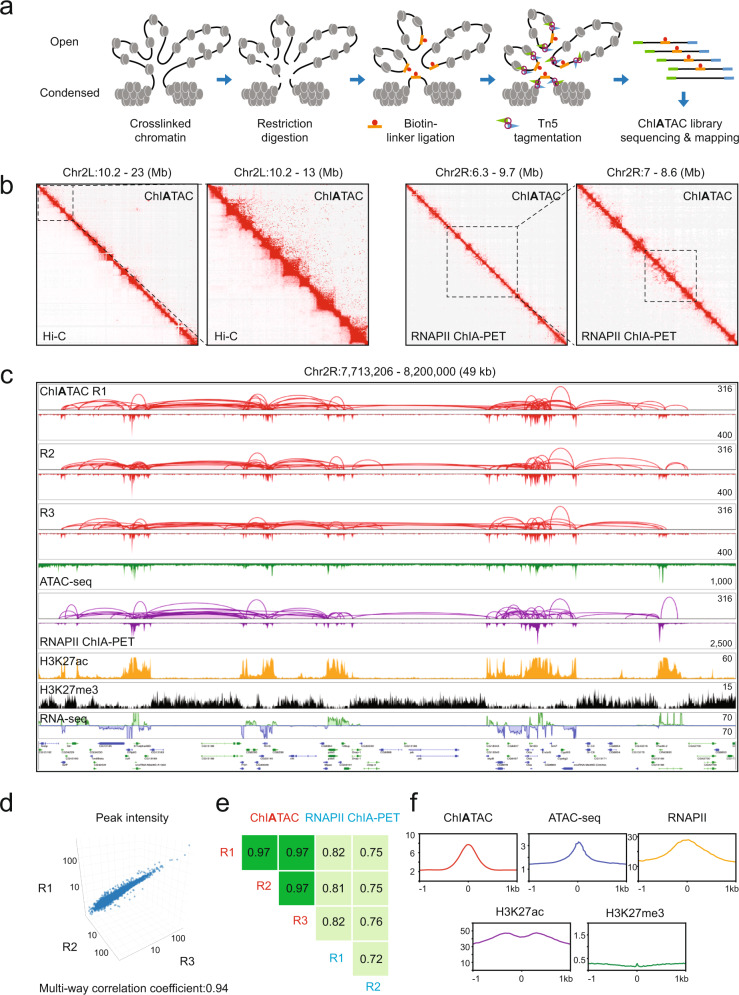
Design of ChIATAC and proof of concept in *Drosophila* cells. **a** ChIATAC schematics. Permeabilized nuclei from crosslinked cells are subjected to in situ restriction enzyme digestion, proximity ligation with biotinylated DNA bridge linker, and then Tn5 tagmentation. Biotinylated DNA ligation fragments with tags are enriched by biotin-streptavidin affinity purification and amplified by PCR. The amplicons (ChIATAC library) are subjected to DNA sequencing and mapping analysis. **b** 2D contact matrices of ChIATAC (top triangle) vs. Hi-C/RNAPII ChIA-PET (bottom triangle) data of *Drosophila* S2 cells at different resolutions. **c** Example view of genome browser tracks of chromatin interaction loops and open chromatin peaks from three replicates of ChIATAC. Tracks of ATAC-seq, RNAPII ChIA-PET (loops and peaks), ChIP-seq of H3K27ac, H3K27me3, and RNA-seq are included to flesh out the broader epigenomic landscape. The signal intensity scales (*y*-axis) of peaks (maximum of reads pileup) and loops (maximum of PET counts) for each track are provided. **d** Reproducibility assessment of peak intensity from three ChIATAC replicates in a 3-way scatter plot. A multi-way correlation coefficient between three replicates is 0.94 (Methods). **e** Genome-wide reproducibility assessment of chromatin interaction data from three replicates of ChIATAC and two replicates of RNAPII ChIA-PET using HiCRep (bin size = 10 kb). The correlation coefficients between the corresponding row and column are shown. **f** Average profile plot in the vicinity (±1 kb) of ChIATAC peaks (*n* = 23,759) and associated epigenomic signals (ATAC, RNAPII, H3K27ac, and H3K27me3) in S2 cells. Source data are provided as a Source Data file.

Our main objective was to develop a method capable of mapping chromatin interactions that reveal transcriptional regulatory programs in samples of a few thousand cells, thereby enabling studies of chromatin interactions to be applied to a broader range of biological and clinical samples. Inspired by the high efficiency and simplicity of the ATAC-seq protocol for mapping open chromatin loci^15^, we designed a strategy for mapping chromatin interactions that combines the proximity ligation of in situ Hi-C^7^ and in situ ChIA-PET^21^ with the Tn5-mediated in situ digestion of ATAC-seq^15^ for simultaneous mapping of chromatin interactions and open chromatin loci with the goal of reducing the required input cells down to a few thousands or even hundreds, without compromising data quality. We call this method ChIA-ATAC, or in short, ChIATAC. In ChIATAC, cells are crosslinked, and the intact but permeabilized nuclei are subjected to in situ restriction enzyme digestion and followed by proximity ligation with a biotinylated bridge linker, as described in the in situ ChIA-PET protocol^21^. The nuclei are then processed for in situ transposase-based tagmentation, as established in the ATAC-seq protocol^15^. The tagged DNA fragments containing chromatin ligation products are isolated by biotin-streptavidin affinity for PCR amplification, and the amplicons (ChIATAC library) are subjected to paired-end-tag DNA sequencing. The sequencing reads are then mapped to the reference genome to reveal chromatin accessibility and interaction (Fig. 1a; Methods).

We first tested a prototype ChIATAC protocol in *Drosophila* Schneider 2 (S2) cells as their smaller genome size facilitates method development. The initial results are summarized in (Supplementary Table 1). The 2D contact matrices of the ChIATAC data matched well with in situ Hi-C and RNA polymerase II (RNAPII) enriched ChIA-PET data generated from the same cells (Fig. 1b and Supplementary Fig. 1a), indicating that ChIATAC successfully captured the overall chromatin folding architecture of S2 cells. We next examined the ChIATAC data at the sub-megabase scale of chromatin domains using genome browser^22^ and found abundant ChIATAC-mapped ‘peaks’ and ‘loops’ anchored at the peak sites (Fig. 1c, Supplementary Fig. 1b). Importantly, we performed three replicates and found that the ChIATAC data were highly reproducible in mapping chromatin 2D contact profiles, loops, and peaks (Fig. 1c–e, Supplementary Fig. 1b–d).

To characterize peaks and loops mapped by ChIATAC, we compared the ChIATAC data with ATAC-seq and RNAPII ChIA-PET data generated from the same S2 cells. We found that the ChIATAC peaks were remarkably comparable to ATAC-seq peaks, and many of them also overlapped with RNAPII ChIA-PET peaks with minor variation in terms of location and intensity (Fig. 1c, Supplementary Fig. 1b). These results confirm that ChIATAC is able to faithfully detect open chromatin loci within the same spectrum as ATAC-seq. We anticipate that many of these sites harbor functional elements involved in transcriptional regulation, as previously suggested^23^. In addition, chromatin loops identified by ChIATAC were largely comparable to those identified by RNAPII ChIA-PET (Fig. 1c, Supplementary Fig. 1b). Indeed, strong chromatin loops and high-intensity peaks in the ChIATAC data were highly associated with transcriptionally active regions, as denoted by the active histone mark H3K27ac, and inversely correlated with the repressive mark H3K27me3 (Fig. 1f, Supplementary Fig. 1e). Taken together, our results suggest that ChIATAC can capture chromatin interactions occurring between open chromatin loci, many of which are anticipated to reflect transcriptional activity across the genome. The fact that ChIATAC largely recapitulates RNAPII ChIA-PET is noteworthy because RNAPII ChIA-PET includes a ChIP-enrichment step, in which an antibody against RNAPII enriches for chromatin interactions associated with transcription, whereas no such ChIP-enrichment step is required for ChIATAC. The simplicity of the ChIATAC protocol is critical for improving the overall efficiency of chromatin interaction analysis for small numbers of input cells. Notably, the *Drosophila* RNAPII ChIA-PET data presented here were generated from 10 million (10^7^) S2 cells, while only 50 thousand (5 × 10^4^) cells were used in ChIATAC, representing a 200-fold reduction of input cells for chromatin interaction analysis. Importantly, the data quality in terms of chromatin contact frequency and peak intensity derived from ChIATAC data were largely comparable with the data of RNAPII ChIA-PET (Fig. 1c, Supplementary Fig. 1b).

### Optimization of ChIATAC for mapping the human 3D epigenome

The successful proof of concept using *Drosophila* S2 cells encouraged us to further optimize the ChIATAC protocol in human cells. The large size and complexity of the human genome impose greater technical challenges than the smaller *Drosophila* genome. Thus, we chose GM12878 cells to test the protocol because this cell line has been well-studied for genomics including 3D genome mapping^7,10,24–27^, and systematically streamlined all major steps in the ChIATAC protocol, including the choice of restriction enzymes for chromatin digestion, titrating the optimal quantity of reagents, and optimizing volumes and temperatures for each reaction (Methods).

For example, we reasoned that the initial restriction digestion and the length of its resulting chromatin fragments would impact^28^ the outcome due to the large size and complexity of the human genome, and we therefore tested the restriction digestion by one enzyme versus two enzymes (Methods). We initially employed AluI (which has a 4-bp recognition site) for in situ chromatin digestion in *Drosophila* cells, where it produced an average chromatin fragment size that plateaued at 5500 bp (Supplementary Fig. 2a). However, when AluI was used for human cells, the fragment size plateaued at 8100 bp. The longer fragment size could limit the resolution and robustness of the final ChIATAC data in mapping chromatin peaks and loops. Thus, we employed a different restriction enzyme, HpyCH4V (also a 4-bp cutter), which produced chromatin fragments of 5500 bp on average. When the two enzymes were combined, the average size of chromatin fragments was reduced to 4600 bp (Supplementary Fig. 2a). To examine the consequences of different fragment lengths, we compared the effects of digestion by AluI (single digestion, SD) or by AluI and HpyCH4V together (double digestion, DD) on ChIATAC library construction, data generation, and mapping. Overall, both SD and DD samples generated robust and reproducible ChIATAC mapping results (Supplementary Table 1, Supplementary Fig. 2b, c). However, the SD ChIATAC data appeared to miss some open chromatin loci that were identified by both DD ChIATAC and ATAC-seq (Supplementary Fig. 2d, e). Furthermore, even though the higher-order chromatin contacts profiled by both datasets appeared similar, DD ChIATAC data captured more detailed chromatin interactions between open chromatin loci as shown in the genome browser view (Supplementary Fig. 2f), likely owing to shorter chromatin fragments enabling better mapping resolution. Therefore, we included double digestion in the final version of the ChIATAC protocol. Lastly, to ensure technical artifacts were not introduced by the ChIATAC protocol, we also performed ChIATAC using purified naked genomic DNA as an input control to provide general background (Supplementary Fig. 2f), which also helped to avoid mapping artifacts in repetitive regions and confirmed that the ChIATAC-identified peaks and loops are bona fide signals.

### Characterization of human ChIATAC data

**Fig. 2:**
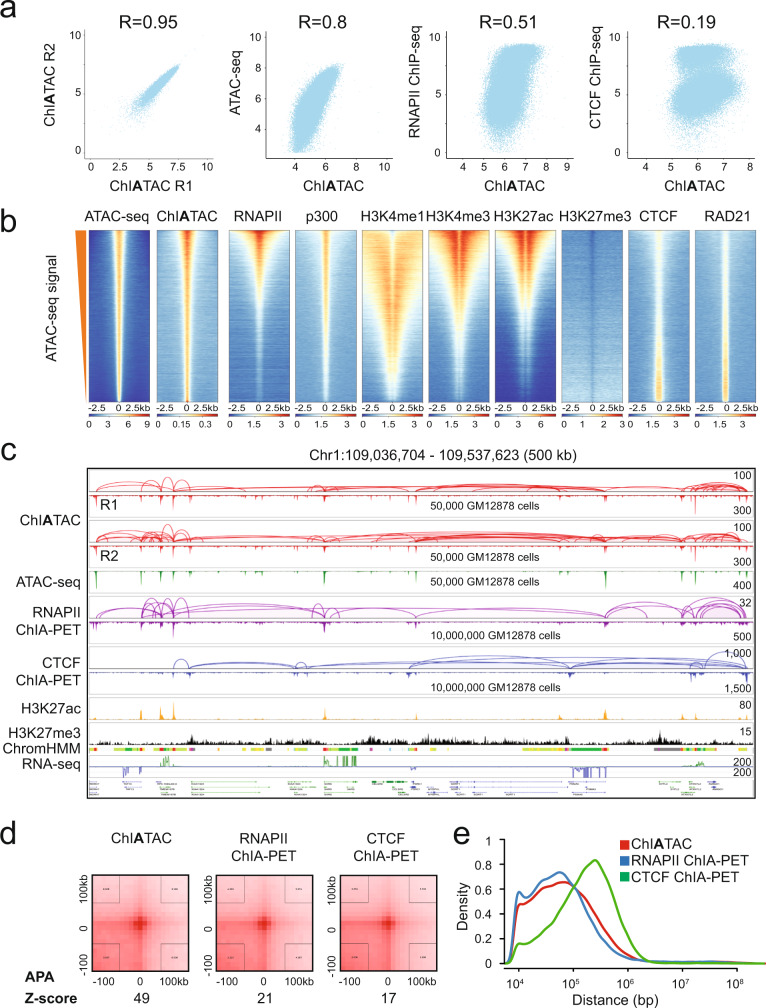
Characterization of human ChIATAC data. **a** Scatter plots of the peak intensity between different datasets at ChIATAC peak (*n* = 71,504) loci. The R-value is the Spearman’s correlation coefficient. **b** Heatmap signals of open chromatin sites (±2.5 kb genomic regions) measured by ATAC-seq and ChIATAC data, and their association with other epigenomic features including transcription active marks (RNAPII, p300, H3K4me1, H3K4me3, and H3K27ac), repressive mark (H3K27me3), and chromatin architectural proteins (CTCF, RAD21). ATAC-seq data were sorted in descending order based on intensity, and the ATAC-seq loci were used as the reference for sorting ChIATAC data. The ChIATAC loci were then sorted in descending order and used as the reference for all other datasets. **c** Example view of genome browser tracks of ChIATAC (SD), ChIATAC (DD), ATAC-seq, RNAPII ChIA-PET, CTCF ChIA-PET, ChIP-seq of H3K27ac, H3K27me3, ChromHMM (red for active promoter, orange for strong enhancer, yellow for weak/poised enhancer, and green for transcribed region), and RNA-seq. The signal intensity scales (*y*-axis) of peaks (maximum of reads pileup) and loops (maximum of PET counts) for each track are provided. **d** The Aggregate Peak Analysis (APA) of chromatin loops in ChIATAC data and the corresponding loop signals in RNAPII ChIA-PET and CTCF ChIA-PET contact matrices. Z-score measures the enrichment of the aggregated signal on the 2D contact matrices. **e** Profiling of intra-chromosomal loop spans of ChIATAC (*n* = 108,191), RNAPII ChIA-PET (*n* = 65,697), and CTCF ChIA-PET (*n* = 58,633) data in GM12878 cells.

Besides showing the high reproducibility (Spearman’s correlation = 0.95) of ChIATAC peaks between two replicates (Fig. 2a), we found a high correlation (Spearman’s correlation = 0.8) between ChIATAC and ATAC-seq in peak intensity (Fig. 2a), confirming similar identification of open chromatin by both methods. Interestingly, we also found a medium-level correlation (Spearman’s correlation = 0.51) between ChIATAC and RNAPII ChIP-seq peaks (Fig. 2a). However, the ChIATAC peaks and CTCF-seq peaks correlated poorly (Spearman’s correlation = 0.19) (Fig. 2a), indicating that ChIATAC-mapped chromatin loci correlate better with RNAPII-associated transcriptional property than CTCF-organized chromatin architecture.

We then systematically characterized the ChIATAC peaks through comparative analysis with available epigenomic data in GM12878 cells generated in the ENCODE project^29^. Indeed, ChIATAC faithfully captured genome-wide open chromatin loci identified by ATAC-seq (Fig. 2b), moreover, the majority of ChIATAC peaks encompassed RNAPII and p300 co-activator binding signals, which are often present at active gene promoters. The peaks also strongly correlated with active transcriptional regulatory elements, as denoted by active histone marks (H3K27ac, H3K4me1, and H3K4me3), and inversely correlated with the repressive mark (H3K27me3). Intriguingly, ChIATAC peaks only partially overlapped with CTCF and RAD21 (a subunit of cohesin) binding sites, and the peak intensity appeared to be inversely correlated (Fig. 2b). To confirm this observation, we performed additional analysis of the ATAC-seq (open chromatin loci, *n* = 75,733) and CTCF ChIP-seq (CTCF binding sites, *n* = 51,014) data. Approximately half (53%, *n* = 27,258) of the CTCF binding sites overlapped with open chromatin sites, while the rest (47%, *n* = 23,756) did not (see details in “Methods”, Supplementary Fig. 2g), presumably reflecting the multifaceted properties of CTCF in association with both active and repressive domains in the genome. It is also noteworthy that there are significant variations in peak intensities at the same loci when mapped using different methods (Supplementary Fig. 2h), reflecting technical differences in the preference of each method for detecting chromatin accessibility (ATAC-seq vs. ChIATAC), protein factor occupancy (ChIP-seq vs. ChIA-PET), and different protein specificity in association with open chromatin (RNAPII vs. CTCF).

Next, we characterized the chromatin loops in ChIATAC data. By comparative analysis with the available GM12878 datasets, i.e., Hi-C^7^ and ChIA-PET (ChIP-enriched for RNAPII and CTCF)^10^, we showed that ChIATAC captured the same higher-order chromatin architectures and domain structures as detected by Hi-C and ChIA-PET (Supplementary Fig. 3a–d). Zoomed-in views of the mapping data at sub-megabase scale by genome browser revealed abundant chromatin interactions that are specifically anchored at ChIATAC peaks of open chromatin loci (Fig. 2c, Supplementary Fig. 3e). To characterize ChIATAC loops using RNAPII and CTCF ChIA-PET data for detailed features of chromatin interactions, we required that both anchors of a loop must be supported by bona fide peaks to ensure high-quality looping data used in our analysis. As such, we identified 108,191 high-quality ChIATAC loops associated with open chromatin loci. The majority (90%, *n* = 7359 out of 8156) of the RNAPII loops (PET ≥ 15) were found in the ChIATAC data (Supplementary Fig. 3f), while less than half (42%, *n* = 7873 out of 18,921) of the CTCF loops (PET ≥ 15) were enriched by ChIATAC (Supplementary Fig. 3f). Moreover, the contact signals of the ChIATAC loops in RNAPII ChIA-PET data were stronger than in the CTCF ChIA-PET data (Fig. 2d). The distribution curves for the genomic length of chromatin loops showed that the profile of ChIATAC loops much more closely resembled the RNAPII ChIA-PET loops than the CTCF ChIA-PET loops (Fig. 2e). Of the CTCF loops (58%, *n* = 11,048 out of 18,921) not identified as enriched in ChIATAC data, 5062 (27% of all CTCF loops) loops overlapped with open chromatin sites, but the PET counts between two anchors in ChIATAC data were below the threshold we used in our analysis pipeline, and 5986 (31% of all CTCF loops) loops had either no anchors or only one anchor associated with open chromatin sites. Overall, the CTCF loops that matched with ChIATAC loops exhibited higher chromatin contact signals than those CTCF loops that were not enriched in ChIATAC data (Supplementary Fig. 3g).

In addition, the chromatin states of 10% of genomic loci annotated by ChromHMM^30,31^ for GM12878 cells were suggested as active gene promoters. However, 28% of the ChIATAC loop anchors were located at gene promoters, which was 18% above the genome background. This discrepancy may reflect a preference of ChIATAC for enriching active epigenomic features (Supplementary Fig. 3h). Finally, we observed that the level of gene expression is positively correlated with the strength (PET counts) of chromatin loops connecting gene promoter and distal cis-regulatory elements (Supplementary Fig. 3i), indicating the importance of chromatin interactions in transcription activation.

Together, our analyses demonstrates that ChIATAC data enrich for chromatin interactions between open chromatin loci for most transcriptional and some architectural chromatin loops, thus confirming that ChIATAC is an effective tool for comprehensive mapping of the human 3D epigenomes.

### Titration of low input cells for human ChIATAC analysis

**Fig. 3:**
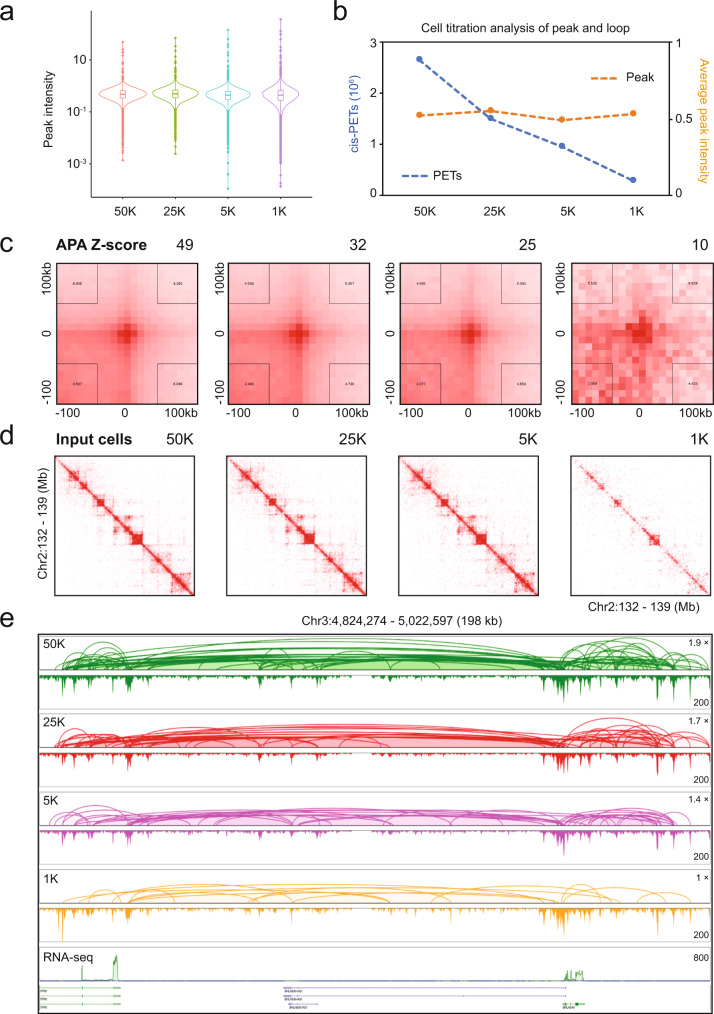
Titration of input cells for ChIATAC analysis. **a** Violin plot of normalized peak intensity (peaks called by using 50 K ChIATAC data, *n* = 71,504) across data produced from 50,000 down to 1000 cells. In the box plot, middle line denotes median; box denotes interquartile range (IQR); and whiskers denote 1.5× IQR. **b** Line plot showing the overview of the number of non-redundant intra-chromosomal PETs (cis-PETs > 8 kb) per hundred million raw reads and normalized average peak intensity (*n* = 71,504) across data produced from 50,000 down to 1000 cells. **c** APA of chromatin loops in ChIATAC data produced by 50,000 cells, and the corresponding loop signals in 2D contact matrices of ChIATAC data produced from 25,000 down to 1000 cells. Z-score measures the enrichment of the aggregated signal on the 2D contact matrices. **d** Example views of 2D contact matrices from ChIATAC data produced from 50,000 down to 1000 cells. **e** Example views of genome browser tracks of ChIATAC data produced from 50,000 (50 K), 25,000 (25 K), 5000 (5 K), down to 1000 (1 K) input cells, and RNA-seq. The normalized fold change of loop PET count is provided. The relative looping frequency (PET counts) is 1.9-fold for 50 K, 1.7-fold for 25 K, 1.4-fold for 5 K, and 1-fold for 1 K input cells. The signal intensity scales (y-axis) of peaks (maximum of reads pileup) for each track are provided. Source data are provided as a Source Data file.

To further investigate the effectiveness of ChIATAC for precious biological samples with limited numbers of cells, we performed a serial titration of ChIATAC analysis with decreased input cells, starting at 50,000 (50 K) and ranging down to 1000 (1 K) cells per ChIATAC library (Methods, Supplementary Table 1). First, we analyzed the ChIATAC peaks of the 4 datasets derived from 50 K, 25 K, 5 K, and 1 K input cells. Based on technical replicates, the ChIATAC peaks derived from more input cells were somewhat more reproducible than the peaks from fewer input cells, as shown by Venn diagrams for overlapping peaks (Supplementary Fig. 4a) and scatter plots for read coverage between replicates (Supplementary Fig. 4b). Also, the correlation among the four titration datasets had a declining trend when input cells were reduced (Supplementary Fig. 4c). However, the median of peak intensity among the four datasets was rather consistent, except that the ChIATAC dataset from 1 K input cells showed wider deviations, with more outlier datapoints (Fig. 3a). Furthermore, we evaluated the local noise flanking each peak and calculated the noise-to-signal ratio (Methods). We observed a similar trend to that of peak intensity, in which the medians of noise-to-signal ratio in the four datasets were similar, but the 5 K and 1 K ChIATAC datasets showed rather greater variation with more outliers (Supplementary Fig. 4d), indicating higher variability of data points. Together, our input cell titration analysis for ChIATAC revealed that although the reduction of input cells could increase sporadic noise, the overall data quality of peaks was largely retained (Supplementary Fig. 4e, f).

Next, we analyzed the chromatin interaction data measured by the PET counts. Like ChIATAC peaks, the chromatin interaction data also showed higher reproducibility with more input cells (50 K and 25 K) than with fewer cells (5 K and 1 K) (Supplementary Fig. 4g). Interestingly, the numbers of ChIATAC chromatin contacts (cis-PETs > 8 kb) steadily declined with decreased input cells in the titration experiments (Fig. 3b), although the average peak intensity of ChIATAC was not significantly impacted by the reduction of input cells (Fig. 3b). Even though the chromatin contact signals showed a substantial decline as measured by APA Z-score^7,32^ along with magnitude reduction of input cells (Fig. 3c) and as exemplified in 2D contact matrices (Fig. 3d), most of the chromatin loops were still distinctively detected by ChIATAC with just 1 K input cells, as shown in detailed genome browser views (Fig. 3e, Supplementary Fig. 4h). Together, our titration experiments demonstrated that as few as 1000 input cells can be used in ChIATAC assay for comprehensive human 3D epigenome mapping.

### Characterization of human 3D epigenomes during T cell activation

Given the robustness of ChIATAC for cultured cells, we sought to apply ChIATAC to primary human cells, with a specific focus on characterizing the 3D epigenome and dynamic changes in primary T cells in response to activation by various extracellular signals. Within the human immune system, it is well established that cell identity and function are largely determined by cell-type-specific transcriptional programs that are regulated by a set of lineage-specific TFs^33^. It has also been suggested that the epigenomic landscape and topological architectures would be altered during T cell differentiation and activation^34^. Although general principles such as the ability of distal enhancers to interact with target gene promoters to activate gene expression have been suggested^5^, the precise topological mechanisms that regulate gene transcription in T cell activation by extracellular signals remain elusive. We were particularly interested in the transformation process of T cell receptor (TCR)-mediated activation of CD4^+^ T cells and subsequent effects of stimulation by IL-2 on the epigenome landscape and the chromatin folding structures, and how such chromatin modifications might lead to altered gene transcription programs in CD4^+^ T cells.

**Fig. 4:**
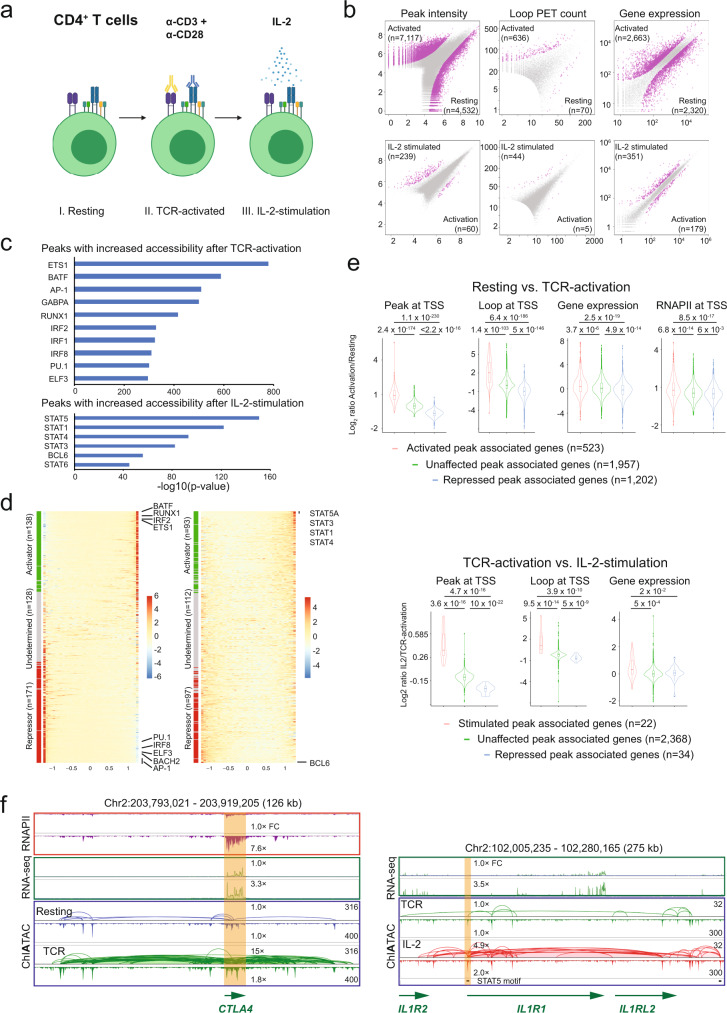
Characterization of human 3D epigenome of T cells in activations. **a** Human CD4^+^ T cells used in ChIATAC experiments: I. Freshly isolated CD4^+^ T cells (resting), II. Cells activated with anti-CD3 + anti-CD28 (TCR-activation). And III. Cells further stimulated with IL-2. **b** Scatter plots showing differential peak intensity (left), chromatin loop PET count (middle), and gene expression (right) between resting vs. TCR-activation (top) and TCR-activation vs. IL-2-stimulation (bottom). The features (peaks, loops, and gene expression) with significant changes are shown as magenta dots, and the numbers of data points are in parenthesis. **c** HOMER motif enrichment analysis from genomic regions with increased chromatin accessibility after TCR-activation (top) and IL-2-stimulation (bottom). **d** Heatmap showing the distribution of the correlations between RNA expression and predicted DNA binding site signal for each TF. TFs are sorted from strongest predicted activator to repressor (from top to bottom). Left, Resting vs. TCR-activation; Right, TCR-activation vs. IL-2-stimulation. **e** Violin plots of chromatin accessibility, looping, gene expression, RNAPII binding at the TSS of genes associated with ChIATAC peaks showing differential chromatin accessibilities after TCR-activation (top) or IL-2-stimulation (bottom). The *p*-values were calculated by the one-sided Wilcoxson rank sum test. In the box plot, middle line denotes median; box denotes interquartile range (IQR); and whiskers denote 1.5× IQR. **f** Example views of genome browser tracks of ChIATAC, RNAPII ChIA-PET, and RNA-seq of CD4^+^ T cells at resting and TCR-activation states (left) or at TCR-activation and IL-2-stimulation states (right). Highlighted are the regions showing increased chromatin accessibility and RNAPII binding after TCR-activation (left) or IL-2-stimulation (right). Relative fold change (FC) of RNAPII binding, RNA-seq, and ChIATAC for looping and chromatin accessibility in reference to the Resting cell data (1.0× FC) are provided. Source data are provided as a Source Data file.

To investigate the steady states of 3D epigenome and dynamic changes in CD4^+^ T cells upon activation, we isolated total CD4^+^ T cells from a buffy coat from a normal blood donor. The CD4^+^ T cells were activated through TCR-activation (anti-CD3 + anti-CD28) for 72 h, rested overnight, and then either not stimulated or stimulated with IL-2 for 4 h (Fig. 4a). We performed RNA-seq and ChIATAC experiments on the three cellular samples (freshly isolated “Resting”, TCR-activated, and IL-2-stimulated following TCR-activation) and generated high-quality data for gene expression, genome-wide mapping of open chromatin loci and chromatin interactions between the loci (Supplementary Fig. 5). We then performed a set of pairwise comparative analyses with the ChIATAC and RNA-seq datasets (Supplementary Table 1) to identify differential chromatin features and transcriptional output. In comparing the ChIATAC data for resting T cells vs. TCR-activated T cells, extensive differences were observed in peaks at corresponding open chromatin loci and chromatin interactions anchored at open chromatin loci (Fig. 4b, top). Specifically, 7117 chromatin loci showed increased chromatin accessibility (higher peak signals in the TCR-activated than in the unstimulated T cells) in the transition from the quiescent state to the activated state, and 4532 chromatin loci were less accessible (lower peak signals in the TCR-activated than in resting T cells). To measure if such changes (intensity increase or decrease) in chromatin accessibility correlated with changes in chromatin connectivity, we examined the peak-associated PET counts (a proxy for assessing the chromatin connectivity associated with the locus) in different conditions (resting vs. TCR-activation). The majority (*n* = 6330; 89% of 7117) of the loci induced by TCR-activation exhibited increased PET counts (Supplementary Fig. 6a); conversely, 88% (*n* = 3989) of the 4,532 loci repressed by TCR-activation showed decreased PET counts (Supplementary Fig. 6b). A similar trend was observed when comparing TCR-activation vs. IL-2-stimulation, in which 97% (*n* = 233) of the activated open chromatin loci after IL-2-stimulation correlated with increased PET counts (Supplementary Fig. 6c), and 97% of the repressed loci by IL-2-stimulation matched with decreased PET counts (Supplementary Fig. 6d). These results reveled that chromatin accessibility are highly correlated with chromatin connectivity. After all, we identified 636 high-quality chromatin interaction loops that were considered activated with significantly increased chromatin contact frequency (PETs) in the TCR-activated cells, while only 70 loops had decreased PETs due to TCR treatment.

By analyzing the corresponding RNA-seq data, we found large numbers of genes that were differentially expressed, with 2663 genes upregulated and 2320 downregulated upon TCR-activation. Together, our data analysis showed substantial modification of chromatin accessibility and long-range chromatin looping as well as altered gene expression in CD4^+^ T cells after TCR-activation.

To further validate the T cell ChIATAC data, we generated ATAC-seq data (Supplementary Table 1) from TCR-activated CD4^+^ T cells and confirmed that the peaks of the TCR-activation ChIATAC data demarcated the same open chromatin loci as ATAC-seq (Supplementary Fig. 7a). We also compared the TCR-activation ChIATAC data with the Trac-loop data also derived from TCR-activated CD4^+^ T cells^18^. As shown (Supplementary Fig. 7b–d), both Trac-loop and ChIATAC data were comparable in capturing chromatin interactions between open chromatin loci, thus validating each other. However, it is noteworthy that Trac-loop required 10^7^−10^8^ cells per experiment^18^, while only 5 × 10^4^ or fewer cells were required for ChIATAC, representing approximately a 1000-fold difference. Moreover, the chromatin interactions in Trac-loop data were mostly (85%) short (<8 kb), and only 15% were long-range contacts (>8 kb); whereas the majority (62%) of chromatin interaction loops in ChIATAC data were long-range (Supplementary Fig. 7e). Further analysis of long-range chromatin interaction loops (>8 kb) revealed that the Trac-loop data predominantly peaked at ~10 kb, whereas the ChIATAC data were mainly in the 80–200 kb range and extended up to 1 Mb (Supplementary Fig. 7f). The predominant short-distance in Trac-loop data may be due to the short-armed nature of the tetramer complex of Tn5, which is used in the method^18^. Thus, the ChIATAC data provided more transcriptional loops over the gene body and long-range interactions between gene promoters, enhancers, and super-enhancers, similar to the RNAPII ChIA-PET data (Supplementary Fig. 7f). We further benchmarked ChIATAC with another method, HiCAR^20^, that also detects open chromatin loci and associated chromatin loops. ChIATAC was not only more robust in performance and required much fewer input cells (1 × 10^3^) than HiCAR (3 × 10^4^), but the quality of the ChIATAC data was also more robust than the HiCAR data. Specifically, the peaks of open chromatin loci identified by ChIATAC were much stronger than those identified by HiCAR, and the open chromatin loops in ChIATAC data revealed more detailed looping structures between active gene promoters and enhancers than were observed in the HiCAR data (Methods; Supplementary Fig. 8).

We also evaluated different computational tools to call chromatin interaction loops in ChIATAC data. There are two different strategies for calling of chromatin loops, one is bin-based (e.g., HICCUPS^32^ and Mustache^35^) and the resolution of loops is dependent on the binning size (e.g., 10 kb or 5 kb); the other one is peak-based (e.g., ChIA-PIPE^22^) if the loop anchors are also experimentally enriched as peaks. We compared ChIA-PIPE (originally developed for ChIA-PET data to simultaneously map TF binding peaks and chromatin interaction loops between the peaks) and HICCUPS (a popular tool for Hi-C data) as well as Mustache (recently developed for Hi-C and Micro-C data) for calling loops in ChIATAC data (see Methods). As shown (Supplementary Fig. 9), ChIA-PIPE performed better than HICCUPS and Mustache, probably due to the nature that the chromatin loops in ChIATAC data are anchored at enriched peaks of open chromatin loci, and that the peak-based loop calling has higher resolution and specificity.

When TCR-activated CD4^+^ T cells were further stimulated by IL-2, only 299 chromatin loci exhibited differential chromatin accessibility, the majority (*n* = 239) of which showed increased peak signals, while only 60 of them had lower peaks (Fig. 4b, bottom). Correspondingly, 44 chromatin loops became stronger, and just 5 loops weakened. In addition, 530 genes were differentially expressed (351 upregulated and 179 downregulated). The relatively minor differences in chromatin remodeling caused by IL-2-stimulation were not obvious in 2D contact matrices but were readily displayed in the genome browser view for peaks and loops (Supplementary Fig. 7g). Interestingly, the 3D epigenomic landscape change during the transition from the quiescent state to the TCR-activated state was a magnitude larger than the change induced by IL-2 (Fig. 4b).

To further investigate which TFs have access to the open chromatin loci with increased chromatin accessibility due to TCR-activation and subsequent IL-2-stimulation, we performed HOMER^36^ motif enrichment analysis (Methods). The most enriched TF motifs after TCR-activation in CD4^+^ T cells include ETS1 (a TF plays essential roles in T cell development and function^37^) and a range of well-known pioneering factors such as BATF, AP-1, and PU.1 (Fig. 4c, top), which have a major impact on shaping the epigenomic landscape by converting chromatin domains from the closed state to open state and lead to recruitment of TFs and transcriptional activation^34^. In contrast, the TF motifs associated with IL-2-stimulation were more limited to STAT family proteins (Fig. 4c, bottom), as previously reported^34^. Specifically, STAT5 is known to be activated by IL-2-stimulation via interaction with the IL-2 receptor to initiate STAT5-regulated transcription^34^.

To explore the possible mode of action of TFs in immune response, we performed diffTF^38^ analysis (Methods) to assess the differential chromatin accessibility and gene expression during the cell-state transition, and further predicted TFs either as putative activators or repressors. Out of the 734 TFs in the diffTF database, 437 TFs showed (Resting vs. TCR-activation) significant differential chromatin accessibility at predicted TFBSs and measurable gene expression. Among them, 138 were classified as activators, 171 as repressors, and 128 were undetermined (Fig. 4d). Similarly, from TCR-activation to IL-2-stimulation, 93 were assigned as activators, 97 as repressors, and 112 were undetermined (Fig. 4d). Among the 20 most significant activators and repressors (activators with the most positive correlations and repressors with the most negative correlations) (Supplementary Data 1), many of them (e.g., ETS1, BATF and well-known TFs involved in immune response such as RUNX1, and IRF2) were also identified by HOMER software (Fig. 4c). BACH2, a TF that has been reported to restrain T cell activation at steady-state^39^, was classified as a putative transcriptional repressor, whereas as expected, STAT5A, STAT3, STAT1, and STAT4 were classified as transcriptional activators (Fig. 4d), as expected. We also noticed that BCL6, a lineage-defining transcription factor for T follicular help cell (T_FH_) cell differentiation^40^, was predicted to be a putative repressor in response to IL-2 (Fig. 4d). The predicted DNA binding sites for BCL6 showed a significant increase in accessibility while the BCL6 gene expression was greatly reduced after IL-2 stimulation. A previous study has shown that IL-2 negatively impacts BCL6 expression, and IL-2 is required for Th9 differentiation^41^. BCL6 inhibits *IL9* gene expression, which encodes a γ_c_-family cytokine Interleukin-9 (IL-9), impairing Th9 differentiation^41^. Our results suggest a potential epigenetic mechanism as to how IL-2 can affect the Th9 cell differentiation by inhibiting the BCL6. We were also able to identify TFs that have not been reported to be involved in the immune response, and our analysis implicates them as putative activators or repressors (Supplementary Data 1).

Next, we investigated how the open chromatin loci with increased chromatin accessibility and chromatin looping after TCR-activation may impact gene transcription programs in CD4^+^ T cells. Of the 7,117 open chromatin loci activated by TCR-activation (Fig. 4b, top), 879 were located at the TSS (promoter) of 523 genes. Such open chromatin loci at gene TSS may directly impact the chromatin looping structure at the gene promoter and thus influence gene expression. We examined the corresponding chromatin loops connecting to the 523 genes and showed that the chromatin contact frequency, as measured by PETs, was significantly increased as compared to other genes in that their chromatin accessibilities were not changed or repressed upon TCR-activation (Fig. 4e, top). At the same time, genes associated with the activated peaks also showed a significant increase of RNAPII binding at their promoters. This trend of epigenomic changes was also observed in gene transcription: the transcription of genes associated with the differential peaks (increased chromatin accessibility) in TCR-activated cells was increased significantly compared to the transcription of genes associated with unaffected (no change in chromatin accessibility) or repressed (decreased chromatin accessibility) loci (Fig. 4e, top). For example, *CTLA4*, a gene that encodes a protein receptor as an immune receptor^42^, was upregulated after TCR-activation. A significant increase in chromatin accessibility was observed at *CTLA4*’s promoter along with a substantial increase in RNAPII occupancy (Fig. 4f, left). The chromatin interactions between the promoter and putative distal cis-regulatory elements were remarkably enhanced after TCR-activation. This example highlights an association of increased chromatin accessibility and chromatin interactions with upregulation of gene expression during TCR-activation in CD4^+^ T cells.

Subsequently, we performed a similar analysis for cells that were stimulated with IL-2 after TCR-activation. Of the 239 IL-2-stimulated ChIATAC peaks, 30 of them were located at or proximal to the promoters (TSSs) of 22 genes. Compared to unchanged or repressed chromatin loci, these 22 genes showed a significant increase in chromatin accessibility, chromatin looping, and expression (Fig. 4e, bottom). For example, at the genomic loci for *IL1RL1* (Fig. 4f, right) and *SOSC2* (Supplementary Fig. 7g), two well-known genes involved in cytokine signaling^43,44^, the ChIATAC data showed a significant increase of signals for both chromatin accessibility and chromatin interactions, corresponding to increased gene transcription after IL-2-stimulation. Importantly, some of the increased chromatin loops anchored at GAS (IFN-γ activated site) motifs (Fig. 4f, right and Supplementary Fig. 7g), which are known to be recognized by IL-2 activated STAT5, thus regulating target genes.

Overall, our ChIATAC data provided comprehensive coverage of the 3D epigenome in CD4^+^ T cells and the changes caused by TCR-activation and IL-2-stimulation. Importantly, our initial analysis demonstrated that 3D epigenomic activation in chromatin accessibility and chromatin interaction looping is strongly correlated with the altered gene expression, thus elucidating a likely topological involvement of regulatory mechanism in gene transcription. The rich datasets generated in this study are also a valuable resource for further dissecting the mechanistic understanding of transcriptional regulatory programs of key genes along with the processes of CD4^+^ T cell activation.

### Topological involvement of transcriptional regulation in T cell activation

**Fig. 5:**
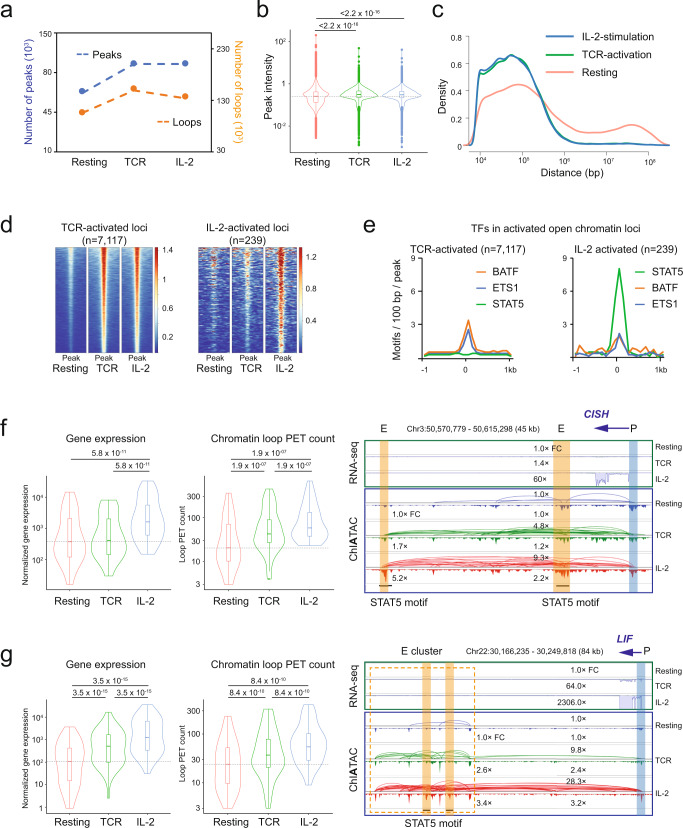
Dynamic changes of T cell 3D epigenome during activations. **a** The numbers of peaks and loops in ChIATAC data of the resting, TCR-activated, and IL-2-stimulated CD4^+^ T cells. **b** Normalized peak intensity of ChIATAC peaks (*n* = 110,466) from three states of CD4^+^ T cells. The *p*-value of significance was calculated by Wilcoxon rank test (one-sided, paired). In the box plot, middle line denotes median; box denotes interquartile range (IQR); and whiskers denote 1.5× IQR. **c** Profiling of intra-chromosomal loop spans in ChIATAC data from three states of CD4^+^ T cells. **d** Heatmaps of peak intensity (±1 kb). Left, TCR-activated open chromatin loci (*n* = 7117) and the corresponding signals in resting and IL-2-stimulated cells. Right, IL-2-stimulated open chromatin loci (*n* = 239) with the signals in resting and TCR-activated cells as references. **e** Aggregation plots for the distribution of TF motifs at open chromatin loci. **f** Group 1 genes (*n* = 34) with moderate change of gene expression after TCR-activation but induced significantly by IL-2-stimulation. Left, violin plots of gene expression and associated loop PET count in three states of CD4^+^ T cells. The dotted lines show the medians in Resting CD4^+^ T cells. The significance was calculated by Wilcoxson signed rank test (one-sided, paired). In all box plots, middle line denotes median; box denotes interquartile range (IQR); and whiskers denote 1.5× IQR. Right, example browser view of RNA-seq and ChIATAC data at enhancer with STAT5 binding motif (highlighted in orange) and gene promoter (highlighted in blue). Relative fold changes (FC) of RNA-seq, loop PET count, and chromatin accessibility in reference to the Resting cell data (1.0× FC) are provided. **g** Group 2 genes (*n* = 48) with significantly induced gene expression and corresponding loop changes by TCR-activation and IL-2-stimulation. Left, same as in **f**, violin plots of gene expression and chromatin loop PET count associated with gene TSSs at 3 states of CD4^+^ T cells. Right, example browser view of RNA-seq and ChIATAC data at enhancer cluster (dotted box) with STAT5 binding motif highlighted in orange and gene promoter highlighted in blue. Same as in **f**, relative fold changes (FC) are provided. Source data are provided as a Source Data file.

To further explore the potential topological involvement of transcriptional regulation in T cells, we investigated the dynamic changes of 3D epigenome during TCR-activation and following stimulation with IL-2. As shown, TCR-activation significantly increased the number of open chromatin peaks and associated chromatin loops, whereas the subsequent IL-2 stimulation had only a minor impact on both peaks and loops (Fig. 5a). The overall peak intensity (or chromatin accessibility) was significantly increased after TCR-activation (Fig. 5b) compared to resting CD4^+^ T cells, perhaps owing to the establishment of new open chromatin regions after TCR-activation, whereas IL-2-stimulation did not result in additional changes (Fig. 5b). Substantial changes in terms of the loop span were also observed after TCR-activation (Fig. 5c). In resting CD4^+^ T cells, many chromatin interactions were in the mega-base range (10^6^−10^7^), and these long-range interactions were not observed after TCR-activation and IL-2-stimulation (Fig. 5c), perhaps reflecting the increased chromatin accessibility and transcriptional chromatin loops in the activated state compared to the resting state with more chromatin compaction. As promoter-enhancer interactions are predominantly found within 1 Mb^25,45^, the disappearance of super long-range interactions (>1 Mb) after TCR-activation may indicate the rewiring of chromatin interactions, which results in a transition from condensed and suppressed chromatin structures to gene-centric chromatin domains with increased chromatin accessibility to TFs and connectivity between promoters and enhancers for activated transcription.

TFs have roles in chromatin remodeling and transcription regulation during the immune response processes^34^. Among the chromatin loci (*n* = 7117) that were more accessible following TCR-activation, the chromatin accessibility was largely maintained in the CD4^+^ cells after subsequent IL-2-stimulation (Fig. 5d, left), whereas fewer loci (*n* = 239) had increased chromatin accessibility specifically stimulated by IL-2 (Fig. 5d, right). Among the top enriched TF motifs in TCR-activated cells, many of them (e.g., ETS1 and BATF) were found with multiple copies near TCR-activated peak loci and maintained the level of motif presence in IL-2 activated peak loci (Fig. 5e); however, in sharp contrast, the STAT5 binding motifs were found specifically in IL-2-activated peaks (Fig. 5e), suggesting the involvement of specific TFs in the transition from resting cells to TCR-activated cells and then to IL-2-stimulated cells.

Together, our pairwise differential (Fig. 4b), TF characterization (Fig. 4c, d), and longitudinal (Fig. 5a–e) analyses all demonstrate that TCR-activation triggered broad and widespread changes in the 3D epigenomic architectures in terms of chromatin accessibility and transcriptional-centric chromatin looping via multiple lineage-specific TFs, which correspond to transcriptional changes of a large number of genes. In contrast, IL-2-stimulation after TCR-activation caused more subtle differences in the 3D epigenomic configuration most likely through the JAK-STAT pathway specifically involving IL-2-activated STAT5.

To understand how IL-2 might induce further differentiation after TCR-activation, we were interested in the genes that are upregulated by IL-2 and their associated chromatin features. We specifically focused on two distinctive gene groups: group 1 (*n* = 34), whose gene expression did not respond to TCR signaling but was specifically induced by IL-2-stimulation (Fig. 5f, left), and group 2 (*n* = 48), which showed a progressive increase of gene expression after TCR-activation and IL-2-stimulation (Fig. 5g, left). However, the examination of the ChIATAC datasets of the three cellular states showed a progressive increase of 3D epigenomic features (loops and peaks) around the genes in both groups. An example of the group 1 genes is *CISH* (encoding cytokine-inducible SH2 containing protein that functions as a suppressor of cytokine signaling^46^), a gene that is potently induced by IL-2. Indeed, our analysis showed that *CISH* specifically responded to IL-2 (60-fold) as measured by RNA-seq but marginally and insignificantly (1.4-fold) to TCR-activation (Fig. 5f, right). Intriguingly, the chromatin interactions in the data from both the ChIATAC TCR-activated and IL-2-induced CD4^+^ T cells showed remarkable increases (4.8-fold by TCR-activation and 9.3-fold by IL-2) in a stepwise and progressive manner, probably due to a sharp surge (5.2-fold) of chromatin accessibility induced by IL-2 at a distal STAT5-bound enhancer more than 40 kb downstream (Fig. 5f, right), suggesting a unique and predominant effect by IL-2-stimulation, but also implying a possible priming phenomenon caused by TCR-activation before IL-2-stimulation. Another example of the group 2 genes is *LIF* (leukemia inhibitory factor). It encodes a protein that is involved in the induction of hematopoietic differentiation in normal and myeloid leukemia cells and is known to be induced by IL-2 significantly^47^. The expression of *LIF* in CD4^+^ T cells was increased 64-fold by TCR-activation and was further induced over 2000-fold by IL-2 (Fig. 5g, right). The ChIATAC peaks at the *LIF* promoter site exhibited 2.4- and 3.2-fold increases of chromatin accessibility by TCR-activation and IL-2-stimulation, respectively; and, remarkably, the chromatin interactions between the *LIF* promoter and an associated enhancer cluster ~80 kb downstream increased by 9.8-fold by TCR-activation and 28.3-fold by IL-2-stimulation, respectively (Fig. 5g, right). It is noteworthy that two of the enhancer sites have STAT5 binding motifs (Fig. 5g, right), helping to explain the IL-2-inducibility of the gene. This example highlights a stepwise and progressive increase in chromatin accessibility and chromatin interactions that correspond to the gene transcription of the target gene.

**Fig. 6:**
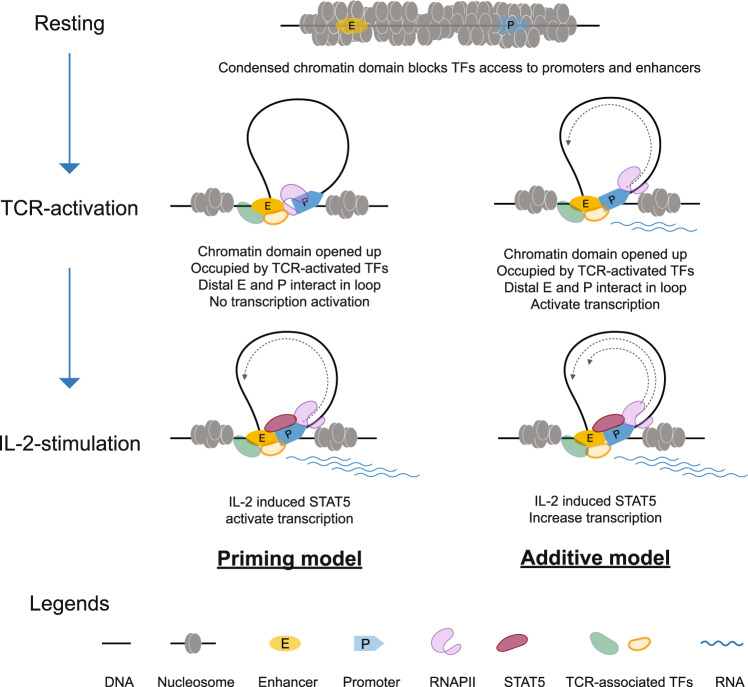
Proposed topological models for IL-2-specific transcription regulation. Schematic illustration of the proposed two topological models for gene transcription activation stimulated by IL-2 following TCR activation in CD4^+^ T cells. In resting stage of CD4^+^ T cells, the chromatin domains harboring IL-2-specific genes may be in a condensed form that suppresses gene transcription. After TCR-activation, the chromatin domains may be remodeled by several pioneering TFs and opened up to be accessible to RNAPII and co-activators. While some of the genes would be activated for transcription, others could be primed with increased potent but not sufficient yet for transcription. After IL-2 stimulation, the poised promoters could be activated for transcription with the participation of STAT5 (the priming model), and the genes that were already activated for transcription with TCR-activation could be further enhanced for transcription with the participation of STAT5 (the additive model).

Thus, although the two groups of genes exhibited different transcriptional outcomes upon TCR-activation, the responses in remodeling of chromatin architectures at the corresponding genomic loci triggered by TCR-activation were similar: stepwise and progressive increase of chromatin accessibility and long-range chromatin connectivity. This phenomenon may also suggest further detailed topological models for the regulation of these two groups of genes—a priming model and the additive model (Fig. 6). In the priming model, the condensed chromatin domains became accessible during TCR-activation, presumably by lineage-specific pioneering factors. The exposed gene promoters and associated enhancers in distance were then brought into proximity to form chromatin loops in a fashion mediated by chromatin architectural proteins and may be occupied by general TFs including RNAPII, however. This resulted in a poised state that was not yet sufficient to activate transcription. After IL-2-stimulated STAT5 activation and DNA binding, transcription of the poised genes is triggered. In the additive model, TCR-activation would be sufficient to activate the gene transcription and is further enhanced by IL-2-mediated activation of STAT5.

## Discussion

Here, we have developed and optimized ChIATAC for simultaneously and comprehensively mapping open chromatin loci and chromatin interactions genome-wide with high accuracy and efficiency from only a few thousand input cells. We established the robustness of ChIATAC using the model systems of *Drosophila* S2 cells and human GM12878 cells. We then demonstrated the application of ChIATAC for comprehensive characterization of the changing 3D epigenome in primary human T cells during ex vivo activation, from the quiescent resting state of CD4^+^ T cells to a TCR-activated state, as well as the following stimulation with IL-2, an important natural activation course occurring in the human immune response to antigen.

The most critical technical advance in ChIATAC is the incorporation of the transposase-based in situ chromatin fragmentation and sequencing adaptor insertion in one reaction step for highly improved efficiency in chromatin DNA library construction, as previously demonstrated in ATAC-seq^15,48^. This modification integrated with chromatin interaction analysis in ChIATAC substantially simplified the protocol, reduced hands-on time, and dramatically improved the efficiency of molecular manipulations, thus successfully achieving the generation of high-quality chromatin interaction data from only thousands of cells using ChIATAC as opposed to the millions of cells that are required when using traditional methods (Hi-C, ChIA-PET, etc.). Another advantageous feature of ChIATAC is its selective enrichment for transcription regulatory interactions between gene promoters, enhancers, and super-enhancers without the ChIP step as is used in ChIA-PET, HiChIP, and PLAC-seq. This is mostly because ChIATAC robustly captures chromatin interactions between open chromatin loci analogous to ATAC-seq for highly efficient mapping of open chromatin loci, and most open chromatin loci are highly associated with active transcription^23^. Lastly, because the critical experimental steps including restriction digestion followed by proximity ligation and transposase fragmentation plus sequencing adaptor addition are all performed in situ without breaking up individual cells or nuclei, the ChIATAC method can be further adapted to various single-cell platforms for developing single-cell ChIATAC.

Applying ChIATAC to primary human T cells demonstrated its potential utility to address a broader range of biological questions with clinical bio-samples that have a limited number of cells. From the CD4^+^ T cells isolated from an individual blood donor and their ex vivo activation by TCR-activation and IL-2-stimulation, we explored potential topological mechanisms of transcription regulation by extracellular signals—here the effects of TCR-activation followed by IL-2-stimulation, mimicking what occurs during the immune response. Combining ChIATAC data for 3D epigenomic features and RNA-seq data for transcriptional output, we traced the dynamic changes of chromatin modification of IL-2-specific genes during the cellular transformation from quiescent to cellular activation in CD4^+^ T cells. Our preliminary analyses are consistent with a topological model in which the chromatin structures are activated by TCR and followed by IL-2 for additional activation in terms of increased chromatin accessibility, enhanced chromatin connectivity, and enhanced transcription. This topological model appeared applicable to IL-2 inducible genes: although RNA-seq did not show upregulation in response to TCR-activation alone, but the surrounding chromatin structures were still significantly modified, with increased chromatin connectivity between open chromatin loci. This observation suggests a possible topological explanation for the previously proposed epigenetic priming model, in which an imposed chromatin modification converts a closed state to an open one during cellular differentiation, resulting in a latent epigenetic state that does not immediately lead to increased gene expression^49^, but cells are nevertheless primed for rapid response upon subsequential inductions^50^. Here, our analysis using ChIATAC data provides evidence that epigenetic priming may also represent the establishment of long-range chromatin interactions between the target gene promoters and essential enhancers that are not by themselves yet sufficient to activate the desired transcription. The *CISH* locus best exemplified this epigenetic priming in action, with TCR triggering a significant chromatin modification, resulting in the establishment of a new chromatin interaction between the *CISH* promoter and its enhancer that did not exist in the resting state of CD4^+^ T cells. However, such establishment was not sufficient to activate *CISH* transcription, and additional IL-2-mediated STAT5 activation and binding to the *CISH* enhancer appear to be required to drive *CISH* transcription (Fig. 5f, right).

Overall, ChIATAC is a valuable and versatile method that we predict will allow the extension of the study of chromatin interactions to a broader range of biological systems, including settings involving rare populations of cells that are difficult to study with other approaches. By revealing how changes in chromatin interactions subserve changes in cell behavior, such studies will lead to a more granular understanding of the mechanistic relationship between genome structure and function.

## Methods

### Cell culture

#### *Drosophila* S2 cell line

*Drosophila* Schneider 2 (S2) cells (derived from a primary culture of late-stage *Drosophila melanogaster* embryos) were cultured in Express Five® SFM (ThermoFisher Scientific; 10486025) with 1:100 L-Glutamine (ThermoFisher Scientific; 25030081) at 27 °C.

#### Human GM12878 cell line

This is a B-lymphoblastoid cell line, originally obtained from Coriell Institute for Medical Research. The cells were cultured in RPMI 1640 (ThermoFisher Scientific; A10491), supplemented with 15% fetal bovine serum (ThermoFisher Scientific; 10082147). The cells were cultured at 37 °C, 5% CO_2_, and ambient oxygen levels as recommended by the Coriell Institute of Medical Research.

### Primary T cell preparation

#### Resting CD4^+^ T cell isolation

An anonymized buffycoat was obtained from the Blood Bank in National Institutes of Health. Buffy coat cells were harvested as a by-product of volunteer-donor blood units and were distributed in an anonymized manner. They meet the criteria for exemption from need for informed consent and IRB review as defined in 45CFR46 and their distribution abides by all NIH guidelines for human subjects research. Human CD4^+^ T cells were purified by EasySep Human CD4^+^ T cell isolation kit (StemCell Technology; 17952).

#### TCR-activation

The isolated primary CD4^+^ T cells were cultured in RPMI1640 medium supplemented with 10% FBS/penicillin-streptomycin in a 37 °C incubator and activated by 2 μg/ml of plate-bound anti-CD3 (Biolegend; 317353) and 1 ug/ml of soluble anti-CD28 (Biolegend; 302901) for 72 h.

IL-2-stimulation: The above TCR-activated T cells were rested overnight. Two hundred International Units of IL-2 were added to the cells for 4 h.

### Cell crosslinking

Harvested cells were crosslinked by 2% formaldehyde (Sigma-Aldrich; 47608) for 20 min at room temperature, the reaction was quenched by adding 0.125 M glycine and incubated at room temperature for 10 min. Cell pellets were washed once with DPBS (Gibco; 14190250). The formaldehyde-crosslinked cells were then crosslinked using 2 mM EGS (ethylene glycol bis(succinimidyl succinate)) (ThermoFisher Scientific; 21565) for 45 min at room temperature. Double-crosslinked cells were quenched by 0.125 M glycine again at room temperature for 10 min and washed once with DPBS. Cells were centrifuged at 2500 × *g* and the supernatants were removed. Cell pellets were either used for an experiment directly or first stored at −80 °C (where they are stable for several months).

### ChIATAC

1000 to 50,000 FA-EGS-crosslinked cells were used as starting material to perform the ChI**A**TAC assay. Cells were first lysed by 100 μl 0.1% SDS FA buffer (50 mM HEPES-KOH, pH 7.5, 150 mM NaCl, 1 mM EDTA, 1% Triton X-100, 0.1% Sodium deoxycholate, 0.1% SDS) at 4 °C for 1 h. Cells were spun down and permeabilized by 10 μl 0.1% SDS at RT for 2 h. The reaction was quenched by adding 2.5 μl 20% Triton X-100 and incubated at 37 °C for 20 min. Cells were then in situ digested by AluI (NEB; R0137L) or AluI+HpyCH4V (NEB; R0620L) restriction enzymes at 37 °C for at least 2 h or overnight by adding 2 μl AluI/ 1 μl AluI + 1 μl HpyCH4V, 5 μl 10× Cutsmart buffer (NEB; B7204S), 25.5 μl ddH_2_O. Restriction enzyme digested chromatin DNA was A-tailed by 0.6 μl 1 mM dATP, 1 μl Klenow Fragment (3′→5′ exo-) (NEB; M0212M), and 1 μl BSA (2 mg/ml) (NEB; B9000S) at 37 °C for 1 h. The reaction is stopped by incubating at 65 °C for 20 min. In situ ligation is then performed by the addition of 20 μl 5× Quick ligation buffer (NEB; B6058S), 1 μl T4 DNA ligase (NEB; M0202L), 3 μl 2 ng/μl bridge linker (Forward strand: 5′-/5Phos/CGCGATATC/iBIOdT/TATCTGACT-3′, reverse strand: 5′-/5Phos/GTCAGATAAGATATCGCGT-3′), and 23.4 μl ddH_2_O for at least 4 h at room temperature or overnight at 16 °C. Cells were spun down and washed once with ATAC-RSB buffer with Tween-20 (10 mM Tris-HCl pH 7.4, 10 mM NaCl, 3 mM MgCl_2_, 0.1% Tween-20). Cells were centrifuged at 2500 × *g* for 5 min and resuspended in 50 μl transposition mix (25 μl 2× TD buffer, 6 μl TDE (Illumina; 20034198), 19 μl DPBS). Transposition reactions were incubated at 37 °C with agitation for 1 h. The DNAs were purified using DNA Clean & Concentrator-5 (Zymo; 4013), eluted in 50 μl Buffer EB (Qiagen; 19086), and incubated with streptavidin M280 beads (ThermoFisher Scientific; 11206D) at RT for 1 h at room temperature. Beads were washed five times with 0.5% SDS/2× SSC buffer and twice with 1× B/W buffer (5 mM Tris-HCl, pH 7.5, 0.5 mM EDTA, 1 M NaCl) then resuspended in Buffer EB. DNA was amplified by adding 25 μl NEBNext® High-Fidelity 2× PCR Master Mix (NEB; M0541S), 5 μl Illumina Nextara XT i7 index primer, and 5 μl Illumina Nextara XT i5 index primer with the following cycling conditions: 72 °C for 3 min, 98 °C for 30 s; 9–12 cycles of 98 °C for 10 s, 63 °C for 30 s, and 72 °C for 40 s; 72 °C for 5 min; 4 °C hold. PCR products were purified using 1× Ampure beads (Beckman Coulter; A63881) and size selected with 0.8× Ampure beads. This final library was sequenced using paired-end sequencing (2 × 150 bp) on an Illumina NovaSeq 6000. Fastq files were generated and demultiplexed with the BCL2Fastq (Illumina). A step-by-step protocol is included in Supplementary Note 1.

### In situ ChIA-PET

In situ ChIA-PET libraries with 20 μg antibodies against RNAPII (Biolegend; 920102) or CTCF (ABclonal; A1133) were constructed using 10,000,000 FA-EGS-crosslinked cells from GM12878 cell cultures, following the in situ ChIA-PET protocol^21^. The ChIA-PET libraries were sequenced by paired-end sequencing (2 × 150 bp) on an Illumina NovaSeq 6000.

### ATAC-seq

ATAC-seq was performed following Omni-ATAC protocol^51^. Briefly, 50,000 cells were harvested and lysed in 50 μl ATAC-RSB buffer with NP-40 and Tween-20 (10 mM Tris-HCl, pH 7.4, 3 mM MgCl_2_, 10 mM NaCl, 0.1% NP-40, 0.1% Tween-20) on ice for 3 min, and immediately centrifuged at 500 × *g* for 10 min at 4 °C. The nuclei pellets were washed once with ATAC-RSB buffer with Tween-20 (10 mM Tris-HCl, pH 7.4, 3 mM MgCl_2_, 10 mM NaCl, and 0.1% Tween-20) resuspended in 50 μl of transposition buffer (25 μl of 2× TD buffer, 16.5 μl DPBS, 0.5 μl 1% digitonin, 0.5 μl 10% Tween-20, and 5 μl water, 2.5 μl of TDE) and incubated at 37 °C for 30 min. Transposed DNA was purified with the DNA Clean & Concentrator-5. This final library was amplified by PCR and sequenced by paired-end sequencing (2 × 150 bp) on an Illumina NovaSeq 6000.

### RNA-seq

Total RNA was extracted using the RNeasy Mini Kit (QIAGEN; 74106) from primary total CD4^+^ T cells or CD4^+^ T cells with TCR-activation or IL-2-stimulation. RNA libraries were prepared using KAPA RNA HyperPrep Kits with RiboErase (HMR) (Roche; 08098140702). This final library was sequenced by paired-end sequencing (2 × 100 bp) on an Illumina NovaSeq 6000.

### ChIATAC and ChIA-PET data processing

ChIATAC and ChIA-PET share the same bridge-linker, and final library structure (adapter-tag-bridge-linker-tag-adapter). The ChIATAC data output is also very similar to ChIA-PET data (producing peaks and loops simultaneously). Thus, ChIATAC and ChIA-PET data were processed using the ChIA-PIPE pipeline^22^ and mapped to the human hg38 reference genome. MACS2^52^ was used to call peaks and the peak intensity from MACS2 output was normalized with ‘-SPMR’ option (signal per million reads). Significant loops were first called by ChIASig^53^ with PET ≥ 3 (PET distance > 8 kb) and FDR < 0.05. The loops were further filtered by anchor support; only significant loops with both anchors supported by peaks were retained. Both ChIATAC and ChIA-PET data can be visualized on Juicebox as 2D contact maps by using Juicer tools^32^ v1.22.01 with built-in normalization options (KR, GW_KR) to create ‘.hic’ file. The 2D contact maps were shown after KR normalization.

### RNA-seq data processing

Reads were trimmed using Trim Galore! (https://github.com/FelixKrueger/TrimGalore) to remove adapters and the portion of the reads with low quality (–stringency 3 -q 20 -e .20 --length 15). Trimmed reads were aligned to the hg38 genome HISAT2^54^ v.2.1.0. We used HTSeq^55^ to quantify mapped reads (MAPQ > 30) to protein-coding genes of GENCODE v36, with parameters for reverse strandedness (–s = reverse). Using the transcript quantifications from HTSeq for those genes, we performed differential gene expression analysis with R package DESeq2^56^. Lowly expressed genes with baseMean <10 in DESeq2 were filtered out. Genes with absolute log2 fold change above 1 and FDR < 0.001 were defined as differentially expressed genes.

### ATAC-seq data processing

Reads were trimmed using Trim Galore! (https://github.com/FelixKrueger/TrimGalore) to remove adapters and that portion of the reads with low quality (--stringency 3 -q 30 -e .20 --length 16). Trimmed reads were aligned to the hg38 genome with BWA-MEM^57^. Duplicated reads were removed with gatk MarkDuplicates (Picard) (https://gatk.broadinstitute.org/). We then retained the reads with mapping quality above 30 and filtered out supplementary alignment with samtools flag ‘-F 2048’.

### Multiway-correlation coefficient analysis

We identified 10,565 common peaks among three replicates of ChIATAC in S2 cells and calculate the SPMR values on the peaks. To demonstrate reproducibility among replicates, we calculated a multi-way correlation coefficient^58^ of the peaks’ SPMR.

### Loop span analysis

Loop span was defined as the linear genomic distance between midpoints of loop anchors.

### Reproducibility assessment of chromatin interaction using HiCRep

HiCRep^59^ was used to assess the reproducibility of ChIATAC replicates and ChIA-PET replicates, which computes the stratum-adjusted correlation coefficient (scc) between two contact matrices for every chromosome. We show the chromosome-length weighted average.

### Heatmap for ChIATAC and ATAC-seq peaks

The epigenomic signals from publicly available data at ChIATAC peaks (*n* = 12,721) ± 1 kb genomic regions in *Drosophila* S2 cells and ATAC-seq peaks (*n* = 75,753) ± 2.5 kb genomic in human GM12878 cells were assessed. Heatmap was generated by the output from the function ‘computeMatrix’ in deepTools^60^ v3.5.1.

### Compartment and insulation score analysis

The compartment score and insulation score were assessed by HiCExplorer^61^ v3.5. All contact matrices were first normalized to 0–1 range and corrected by Knight-Ruiz Matrix Balancing (KR). Compartment score is based on PCA1 computed with 200-kb bin size using ‘hicPCA’ function. The insulation score was computed with 25-kb bin size using ‘hicFindTADs’ function.

### Peak noise level analysis

We assessed noise level around a peak by calculating the ratio between SPMR on the peak region and its flanking regions. The flanking regions were composed of 500 bp from the leftmost and 500 bp from the rightmost of the peak region. The noise level is defined as the average SPMR of flanking region divided by that of peak region.

### APA analysis

The Aggregate Peak Analysis (APA) was done by using Juicer tools^32^ v1.22.01 with -KR options at 10,000 bp resolution. The Z-score of the central pixel relative to all the pixels in the lower left (LL) corner was used to examine the peak signal enrichment.

### Differential chromatin accessibility analysis

Differential chromatin accessibility analysis was carried out by using R Bioconductor package Diffbind^62^ v3.4.1. The trimmed mean of M-values approach (TMM) method was used to normalize compositional differences between libraries. To calculate the library size, we sum up the reads that overlap consensus peaks in each sample (Reads in Peaks). Peaks with FDR < 1% and absolute log2 fold change > 1 were defined as differentially accessible regions.

### Differential interactions analysis

Significant loops with PETs > 10 were used for analysis. All loop anchors from two replicates of the comparing conditions were collected, and a list of unique anchors was generated by merging the overlapping anchors into larger regions. These unique anchors were indexed and anchor pairs for each loop were identified. A unique ID was then assigned to a consensus loop. A table that contains PET counts of unique consensus loops was generated for each library. We used DESeq2 to normalize the PET counts of consensus loops from the four libraries being compared and computed the differential loops based on the PET counts. Interactions with absolute log2 FC > 1 and FDR < 0.01 were defined as differential interactions.

### Motif analysis

The genomic regions with increased chromatin accessibility after TCR-activation or IL-2-stimulation were examined for enriched motifs of known transcription factors by using ‘findMotifsGenome.pl’ function in HOMER software^36^ v4.11. Visualizing motif positions and motif distribution analysis was done by using ‘annotatePeaks.pl’ function.

### diffTF analysis

diffTF classifies TFs based on the assumption that increasing the level of an activating TF increases chromatin accessibility at its target sites while increasing the level of a repressing TF decreases it. For each TF, diffTF calculated the Pearson correlation between the expression level of each TF and the chromatin accessibility across all the peaks with predicted TFBS for a particular TF. If the median of the resulting correlation coefficients was sufficiently positive, diffTF considers it an activator, if sufficiently negative as a repressor, and if it was not significantly different from the background, diffTF calls it undetermined. Two replicates of ChI**A**TAC 1D chromatin accessibility and RNA-seq data were used for each condition. Pairwise comparisons using diffTF^38^ between (1) Resting vs. TCR-activation; (2) TCR-activation vs. IL-2-stimulation) were performed. We classified putative transcriptional activators and repressors based on adjusted *p* value < 0.2, stringency = 0.001, and raw *p* value < 0.2, stringency = 0.001 for (Resting vs. TCR-activation) and (TCR-activation vs. IL-2 stimulation), respectively.

### Trac-loop data analysis for comparison with ChIATAC data

Trac-loop data from TCR-activated CD4^+^ T cells were downloaded from Lai et al.^18^ and we used the liftOver utility to convert from hg18 to hg38. The interaction distance between two tags was examined by using PETs from GSM2326181_CD4_Activated_TrAC-Looping_rep1-tech1. PETs up to 1 kb were normalized by reads per million and used to generate Trac-loop one dimensional (1D) coverage track. We filtered out PETs below 1 kb then using the remaining interactions we generated a hic heatmap with Juicer tools v1.22.01^32^. Significant loops from GSE87254_DHS1K_stim_3PETs_fdr were filtered with PET distance > 8 kb and used to generate Trac-loop chromatin interaction track.

### HiCAR data processing for comparison with ChIATAC data

HiCAR is another method that can detect open chromatin loci and chromatin interactions. The HiCAR data from GM12878 cells were downloaded from Wei et al.^20^. The raw data were processed using the Nextflow pipeline (https://nf-co.re/hicar) to get the 1D coverage. The interactions (*n* = 48,516) from Wei et al^20^. were used for characterizing the chromatin loops.

We further benchmarked ChIATAC and HiCAR for both: (i) Peak and intensity of open chromatin loci; (ii) Chromatin interactions among open chromatin sites. The majority (67–70%) of ChIATAC and HiCAR peaks overlapped (Supplementary Fig. 8a), while ChIATAC showed higher peak intensity at the peak loci demarcated by ATAC-seq as compared to HiCAR (Supplementary Fig. 8b, c). Regarding the chromatin interactions, ChIATAC loops showed a similar profile in distribution curves of loop distance with RNAPII ChIA-PET, ranging from 10 to 100 kb with a median of 62,313 bp, whereas HiCAR loops were much longer with a median length of 145,000 bp (Supplementary Fig. 8d). ChIATAC loops showed more refined looping structures (higher resolution) than HiCAR loops (Supplementary Fig. 8e). The discrepancy of loop distance between two methods might result from the different loop calling strategies that were utilized, with ChIATAC using ChIA-PIPE^22^ to cluster PETs in the vicinity and HiCAR using a bin-based loop-calling method, i.e., MAPS^63^. Thus, we further compared several common loop-calling methods, i.e., Mustache^35^ and HICCUPS^32^ (Supplementary Fig. 9). To ensure accurate loop calling, we used in situ Hi-C data in GM12878 cells as a reference, both Mustache (5 kb bin, *n* = 19,322; 10 kb bin, *n* = 13,670) and HICCUPS (5 kb bin, *n* = 7215; 10 kb bin, *n* = 8001) called similar number of loops as reported previously. Eventually, we found that ChIA-PIPE is the most suitable loop-calling method for ChIATAC data.

### HiCCUPS loop calling

HiCCUPS^32^ was used for loop calling in ChIATAC data (bin size: 10 kb and 5 kb) with -k KR -f 0.1 option.

### Mustache loop calling

Mustache^35^ was used for loop calling in ChIATAC data (bin size: 10 kb and 5 kb) with -pt 0.05 -norm KR option.

### Statistics and reproducibility

No statistical method was used to predetermine sample size. No data were excluded from the analyses. The experiments were not randomized.

### Reporting summary

Further information on research design is available in the Nature Portfolio Reporting Summary linked to this article.

## Supplementary information

Supplementary Information

Reporting Summary

Description of Additional Supplementary Files

Supplementary Data 1

## Data Availability

Data are available at GEO under accession number GSE194036. Supplementary table 1 lists all datasets generated and used in this study. Source data are provided with this paper.

## Source data

Source Data

## References

[1] Zheng H, Xie W (2019). The role of 3D genome organization in development and cell differentiation. Nat. Rev. Mol. Cell Biol..

[2] Rowley MJ, Corces VG (2018). Organizational principles of 3D genome architecture. Nat. Rev. Genet..

[3] Bonev B, Cavalli G (2016). Organization and function of the 3D genome. Nat. Rev. Genet..

[4] Kempfer R, Pombo A (2020). Methods for mapping 3D chromosome architecture. Nat. Rev. Genet..

[5] Schoenfelder S, Fraser P (2019). Long-range enhancer–promoter contacts in gene expression control. Nat. Rev. Genet..

[6] Lieberman-Aiden E (2009). Comprehensive mapping of long-range interactions reveals folding principles of the human genome. Science.

[7] Rao SS (2014). A 3D map of the human genome at kilobase resolution reveals principles of chromatin looping. Cell.

[8] Fullwood MJ (2009). An oestrogen-receptor-α-bound human chromatin interactome. Nature.

[9] Li G (2012). Extensive promoter-centered chromatin interactions provide a topological basis for transcription regulation. Cell.

[10] Tang Z (2015). CTCF-mediated human 3D genome architecture reveals chromatin topology for transcription. Cell.

[11] Mumbach MR (2016). HiChIP: efficient and sensitive analysis of protein-directed genome architecture. Nat. Methods.

[12] Fang R (2016). Mapping of long-range chromatin interactions by proximity ligation-assisted ChIP-seq. Cell Res..

[13] Du Z (2017). Allelic reprogramming of 3D chromatin architecture during early mammalian development. Nature.

[14] Zhang K (2020). Analysis of genome architecture during SCNT reveals a role of cohesin in impeding minor ZGA. Mol. Cell.

[15] Buenrostro JD, Giresi PG, Zaba LC, Chang HY, Greenleaf WJ (2013). Transposition of native chromatin for fast and sensitive epigenomic profiling of open chromatin, DNA-binding proteins and nucleosome position. Nat. Methods.

[16] Song L, Crawford GE (2010). DNase-seq: a high-resolution technique for mapping active gene regulatory elements across the genome from mammalian cells. Cold Spring Harb. Protoc..

[17] Giresi PG, Kim J, McDaniell RM, Iyer VR, Lieb JD (2007). FAIRE (Formaldehyde-Assisted Isolation of Regulatory Elements) isolates active regulatory elements from human chromatin. Genome Res..

[18] Lai B (2018). Trac-looping measures genome structure and chromatin accessibility. Nat. Methods.

[19] Li T, Jia L, Cao Y, Chen Q, Li C (2018). OCEAN-C: mapping hubs of open chromatin interactions across the genome reveals gene regulatory networks. Genome Biol..

[20] Wei X (2022). HiCAR is a robust and sensitive method to analyze open-chromatin-associated genome organization. Mol. Cell.

[21] Wang P (2021). In situ chromatin interaction analysis using paired-end tag sequencing. Curr. Protoc..

[22] Lee B (2020). ChIA-PIPE: a fully automated pipeline for comprehensive ChIA-PET data analysis and visualization. Sci. Adv..

[23] Klemm SL, Shipony Z, Greenleaf WJ (2019). Chromatin accessibility and the regulatory epigenome. Nat. Rev. Genet..

[24] Ma W (2015). Fine-scale chromatin interaction maps reveal the cis-regulatory landscape of human lincRNA genes. Nat. Methods.

[25] Mifsud B (2015). Mapping long-range promoter contacts in human cells with high-resolution capture Hi-C. Nat. Genet..

[26] Quinodoz SA (2018). Higher-order inter-chromosomal hubs shape 3D genome organization in the nucleus. Cell.

[27] Tan L, Xing D, Chang C-H, Li H, Xie XS (2018). Three-dimensional genome structures of single diploid human cells. Science.

[28] Akgol Oksuz B (2021). Systematic evaluation of chromosome conformation capture assays. Nat. Methods.

[29] Dunham I (2012). An integrated encyclopedia of DNA elements in the human genome. Nature.

[30] Ernst J, Kellis M (2010). Discovery and characterization of chromatin states for systematic annotation of the human genome. Nat. Biotechnol..

[31] Ernst J (2011). Mapping and analysis of chromatin state dynamics in nine human cell types. Nature.

[32] Durand NC (2016). Juicer provides a one-click system for analyzing loop-resolution Hi-C experiments. Cell Syst..

[33] Hosokawa H, Rothenberg EV (2021). How transcription factors drive choice of the T cell fate. Nat. Rev. Immunol..

[34] Li, P. & Leonard W. J. Chromatin accessibility and interactions in the transcriptional regulation of T cells. *Front. Immunol.* **9**, 2738 (2018). 10.3389/fimmu.2018.02738 PMC6262064 30524449

[35] Roayaei Ardakany A, Gezer HT, Lonardi S, Ay F (2020). Mustache: multi-scale detection of chromatin loops from Hi-C and Micro-C maps using scale-space representation. Genome Biol..

[36] Heinz S (2010). Simple combinations of lineage-determining transcription factors prime cis-regulatory elements required for macrophage and B cell identities. Mol. Cell.

[37] Garrett-Sinha LA (2013). Review of Ets1 structure, function, and roles in immunity. Cell. Mol. Life Sci..

[38] Berest I (2019). Quantification of differential transcription factor activity and multiomics-based classification into activators and repressors: diffTF. Cell Rep..

[39] Richer MJ, Lang ML, Butler NS (2016). T cell fates zipped up: how the Bach2 basic leucine zipper transcriptional repressor directs T cell differentiation and function. J. Immunol..

[40] Choi J, Crotty S (2021). Bcl6-mediated transcriptional regulation of follicular helper T cells (T_FH_). Trends Immunol..

[41] Liao W (2014). Opposing actions of IL-2 and IL-21 on Th9 differentiation correlate with their differential regulation of BCL6 expression. Proc. Natl Acad. Sci. USA.

[42] Waterhouse P (1995). Lymphoproliferative disorders with early lethality in mice deficient in Ctla-4. Science.

[43] Dinarello CA (2018). Overview of the IL-1 family in innate inflammation and acquired immunity. Immunol. Rev..

[44] Minamoto S (1997). Cloning and functional analysis of new members of STAT induced STAT inhibitor (SSI) family: SSI-2 and SSI-3. Biochem. Biophys. Res. Commun..

[45] Nasser J (2021). Genome-wide enhancer maps link risk variants to disease genes. Nature.

[46] Aman MJ (1999). CIS associates with the interleukin-2 receptor β chain and inhibits interleukin-2-dependent signaling. J. Biol. Chem..

[47] Li P (2017). STAT5-mediated chromatin interactions in superenhancers activate IL-2 highly inducible genes: functional dissection of the *Il2ra* gene locus. Proc. Natl Acad. Sci. USA.

[48] Adey A (2010). Rapid, low-input, low-bias construction of shotgun fragment libraries by high-density in vitro transposition. Genome Biol..

[49] Hu G (2018). Transformation of accessible chromatin and 3D nucleome underlies lineage commitment of early T cells. Immunity.

[50] Bevington SL (2016). Inducible chromatin priming is associated with the establishment of immunological memory in T cells. EMBO J..

[51] Corces MR (2017). An improved ATAC-seq protocol reduces background and enables interrogation of frozen tissues. Nat. Methods.

[52] Zhang Y (2008). Model-based analysis of ChIP-Seq (MACS). Genome Biol..

[53] Paulsen J, Rødland EA, Holden L, Holden M, Hovig E (2014). A statistical model of ChIA-PET data for accurate detection of chromatin 3D interactions. Nucleic Acids Res..

[54] Kim D, Langmead B, Salzberg SL (2015). HISAT: a fast spliced aligner with low memory requirements. Nat. Methods.

[55] Anders S, Pyl PT, Huber W (2014). HTSeq—a Python framework to work with high-throughput sequencing data. Bioinformatics.

[56] Love MI, Huber W, Anders S (2014). Moderated estimation of fold change and dispersion for RNA-seq data with DESeq2. Genome Biol..

[57] Li, H. Aligning sequence reads, clone sequences and assembly contigs with BWA-MEM. Preprint at https://ui.adsabs.harvard.edu/abs/2013arXiv1303.3997L (2013).

[58] Taylor, B. M. A multi-way correlation coefficient. Preprint at https://ui.adsabs.harvard.edu/abs/2020arXiv200302561T (2020).

[59] Lin D, Sanders J, Noble WS (2021). HiCRep.py: fast comparison of Hi-C contact matrices in Python. Bioinformatics.

[60] Ramírez F (2016). deepTools2: a next generation web server for deep-sequencing data analysis. Nucleic Acids Res..

[61] Wolff J (2020). Galaxy HiCExplorer 3: a web server for reproducible Hi-C, capture Hi-C and single-cell Hi-C data analysis, quality control and visualization. Nucleic Acids Res..

[62] Ross-Innes CS (2012). Differential oestrogen receptor binding is associated with clinical outcome in breast cancer. Nature.

[63] Juric I (2019). MAPS: Model-based analysis of long-range chromatin interactions from PLAC-seq and HiChIP experiments. PLoS Comput. Biol..

