# Supplementary material for: ChIATAC is an efficient strategy for multi-omics mapping of 3D epigenomes from low-cell inputs
Source: Nat Commun. 2023 Jan 13;14:213. doi: 10.1038/s41467-023-35879-5 (PMC9839710; doi:10.1038/s41467-023-35879-5)
Supplement: Supplementary file 1 — Supplementary Information [file 41467_2023_35879_MOESM1_ESM.pdf]

# Supplementary Table1. Datasets generated and used in this study.

| | | | | | | |
|---|---|---|---|---|---|---|
| **S2 cell** | | | | | | |
| **Biosample** | **Experiment** | **Enzyme digestion** | **Library ID** | **# of reads** | **Accession** | **Data source** |
| S2 | ChIATAC B1T1 (50k) | Alu1 | TDS0007V | 61,299,828 | | This study |
| S2 | ChIATAC B1T2 (50k) | Alu1 | TDS0008V | 72,601,455 | | This study |
| S2 | ChIATAC B1T3 (50k) | Alu1 | TDS0011V | 93,306,475 | | This study |
| S2 | Long read RNAPII ChIA-PET | None | LDS02110210C | 39,113,518 | GSM3347527 | Zheng et al., 2019 |
| S2 | ATAC-seq | | | | GSE119708 | Albig et al., 2019 |
| S2 | RNAPII ChIP-seq | | | | GSM1017403 | Herz et al., 2012 |
| S2 | H3K27ac ChIP-seq | | | | GSM1017404 | Herz et al., 2012 |
| S2 | H3K27me3 ChIP-seq | | | | GSM480157 | Gan et al., 2010 |
| S2 | RNA-seq | | | | ENCSR237JFT | ENCODE |
| S2 | in situ Hi-C | | | | 4DNESFOADERB | Ray et al., 2019 |
| | | | | | | |
| **GM12878** | | | | | | |
| **Biosample** | **Experiment** | **Enzyme digestion** | **Library ID** | **# of reads** | **Accession** | **Data source** |
| GM12878 | ChIATAC B1T1 (50k) | Alu1 | THG0014 | 554,318,822 | | This study |
| GM12878 | ChIATAC B1T2 (50k) | Alu1 | THG0015 | 647,415,117 | | This study |
| GM12878 | ChIATAC B1T1 (50k) | Alu1&Hpy | THG0019 | 804,323,974 | | This study |
| GM12878 | ChIATAC B1T2 (50k) | Alu1&Hpy | THG0027 | 672,753,900 | | This study |
| GM12878 | ChIATAC B1T1 (25k) | Alu1 | THG0016 | 1,647,128,979 | | This study |
| GM12878 | ChIATAC B1T2 (25k) | Alu1&Hpy | THG0020 | 717,654,042 | | This study |
| GM12878 | ChIATAC B1T1 (5k) | Alu1&Hpy | THG0018 | 693,960,013 | | This study |
| GM12878 | ChIATAC B1T3 (5k) | Alu1&Hpy | THG0024 | 926,677,259 | | This study |
| GM12878 | ChIATAC B1T1 (1k) | Alu1&Hpy | THG0025 | 768,000,805 | | This study |
| GM12878 | ChIATAC B1T2 (1k) | Alu1&Hpy | THG0026 | 852,702,475 | | This study |
| GM12878 | ChIATAC input control | Alu1 | THG0036 | 171,432,505 | | This study |
| GM12878 | ChIATAC input control | Alu1&Hpy | THG0037 | 183,280,599 | | This study |
| GM12878 | in situ RNAPII ChIA-PET B1T1 | Alu1 | LHG0035V | 253,204,012 | | This study |
| GM12878 | in situ RNAPII ChIA-PET B1T2 | Alu1 | LHG0045V | 494,494,353 | | This study |
| GM12878 | in situ CTCF ChIA-PET B1T1 | Alu1 | LHG0052H | 358,752,218 | | This study |
| GM12878 | in situ CTCF ChIA-PET B1T2 | Alu1 | LHG0066V | 340,206,563 | | This study |
| GM12878 | in situ Hi-C | | NA | | 4DNFI1UEG1HD | 4DN |
| GM12878 | RNA-seq | | NA | | ENCLB555AQG | ENCODE |
| GM12878 | ATAC-seq | | NA | | ENCFF603BJO | ENCODE |
| GM12878 | H3K27ac ChIP-seq | | NA | | ENCFF340JIF | ENCODE |
| GM12878 | H3K27me3 ChIP-seq | | NA | | ENCFF684XBR | ENCODE |
| GM12878 | H3K4me1 ChIP-seq | | NA | | ENCFF564KBE | ENCODE |
| GM12878 | H3K4me3 ChIP-seq | | NA | | ENCFF919DOR | ENCODE |
| GM12878 | CTCF ChIP-seq | | NA | | ENCFF800WUV | ENCODE |
| GM12878 | RAD21 ChIP-seq | | NA | | ENCFF571ZJJ | ENCODE |
| GM12878 | EP300 ChIP-seq | | NA | | ENCFF482JMC | ENCODE |
| GM12878 | RNAPII ChIP-seq | | NA | | ENCFF203NVD | ENCODE |
| | | | | | | |
| **$CD4^+$ T cell** | | | | | | |
| **Biosample** | **Experiment** | **Enzyme digestion** | **Library ID** | **# of reads** | **Accession** | **Data source** |
| $CD4^+$ Resting | ChIATAC B1T1 (50k) | Alu1&Hpy | THN0017 | 1,714,508,830 | | This study |
| $CD4^+$ Resting | ChIATAC B1T2 (50k) | Alu1&Hpy | THN0018 | 1,585,614,601 | | This study |
| $CD4^+$ TCR-activation | ChIATAC B1T1 (50k) | Alu1&Hpy | THN0009 | 816,888,402 | | This study |
| $CD4^+$ TCR-activation | ChIATAC B1T2 (50k) | Alu1&Hpy | THN0010 | 841,186,355 | | This study |
| $CD4^+$ IL2-stimulation | ChIATAC B1T1 (50k) | Alu1&Hpy | THN0011 | 757,984,579 | | This study |
| $CD4^+$ IL2-stimulation | ChIATAC B1T2 (50k) | Alu1&Hpy | THN0012 | 798,161,777 | | This study |
| $CD4^+$ Resting | rRNA depleted stranded RNA-seq B1T1 | NA | NA | | ENCSR033XWU | ENCODE |
| $CD4^+$ Resting | rRNA depleted stranded RNA-seq B1T2 | NA | NA | | ENCSR411MUF | ENCODE |
| $CD4^+$ TCR-activation | rRNA depleted stranded RNA-seq B1T1 | NA | RHN0009 | 72,414,803 | | This study |
| $CD4^+$ TCR-activation | rRNA depleted stranded RNA-seq B1T2 | NA | RHN0010 | 56,368,600 | | This study |
| $CD4^+$ IL2-stimulation | rRNA depleted stranded RNA-seq B1T1 | NA | RHN0011 | 70,452,934 | | This study |
| $CD4^+$ IL2-stimulation | rRNA depleted stranded RNA-seq B1T2 | NA | RHN0012 | 68,745,227 | | This study |
| $CD4^+$ TCR-activation | ATAC-seq | NA | AHN0005 | 409,238,057 | | This study |
| $CD4^+$ Resting | in situ RNAPII ChIA-PET B1T1 | Alu1 | LHT0057V | 339,159,585 | | This study |
| $CD4^+$ TCR-activation | in situ RNAPII ChIA-PET B1T1 | Alu1 | LHT0040N | 410,067,626 | | This study |
| | | | | | | |
| | | | | | | |
| *B for Biological replicates | | | | | | |
| *T for Technical replicates | | | | | | |

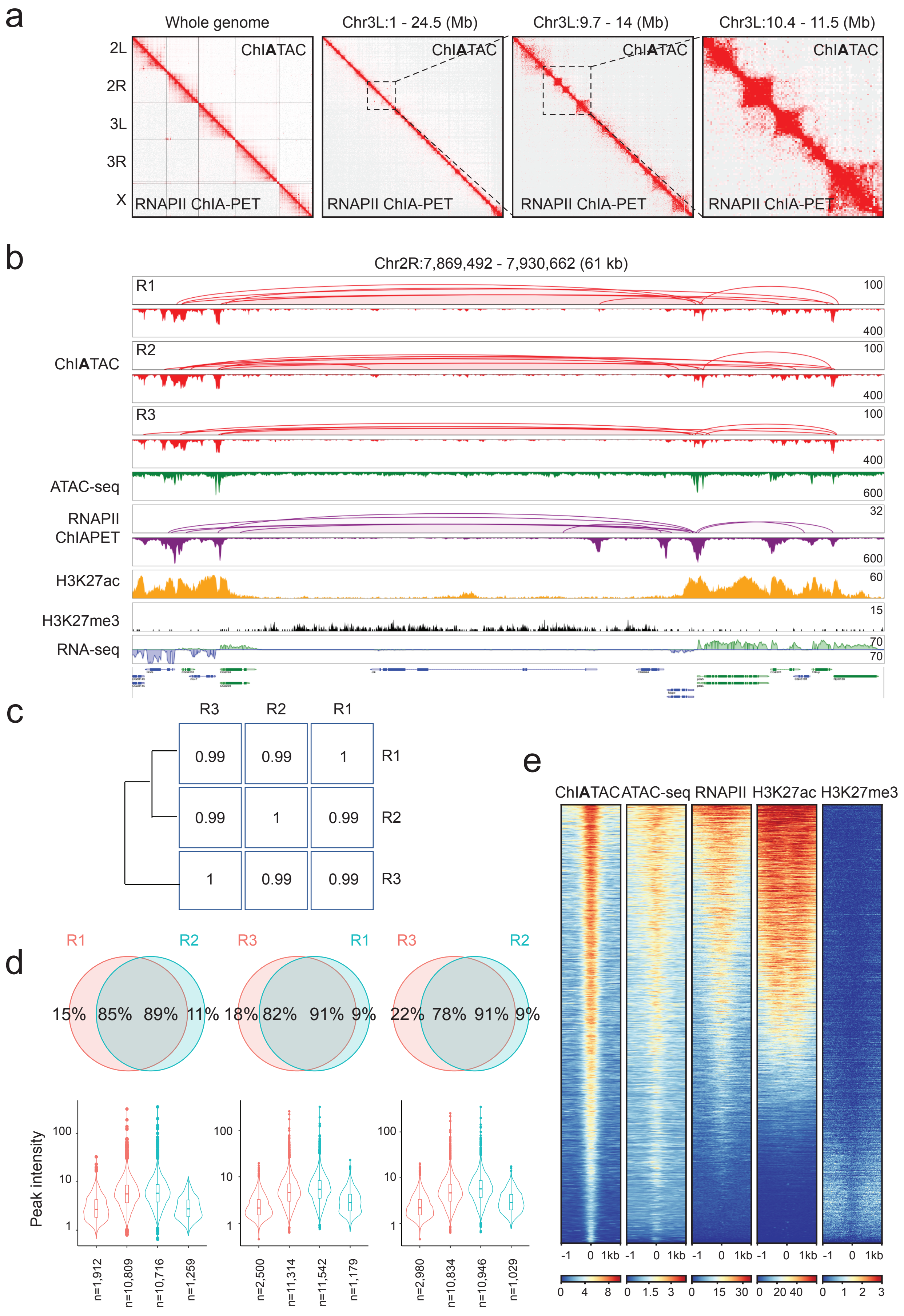

a
Whole genome
Chr3L:1 - 24.5 (Mb)
Chr3L:9.7 - 14 (Mb)
Chr3L:10.4 - 11.5 (Mb)
ChIATAC
RNAPII ChIA-PET
2L
2R
3L
3R
X
b
Chr2R:7,869,492 - 7,930,662 (61 kb)
ChIATAC
R1
R2
R3
100
400
ATAC-seq
600
RNAPII ChIAPET
32
600
H3K27ac
60
H3K27me3
15
RNA-seq
70
70
c
R3
R2
R1
0.99
0.99
1
0.99
1
0.99
1
0.99
0.99
d
R1
R2
15%
85%
89%
11%
R3
R1
18%
82%
91%
9%
R3
R2
22%
78%
91%
9%
Peak intensity
100
10
1
n=1,912
n=10,809
n=10,716
n=1,259
n=2,500
n=11,314
n=11,542
n=1,179
n=2,980
n=10,834
n=10,946
n=1,029
e
ChIATAC
ATAC-seq
RNAPII
H3K27ac
H3K27me3
-1 0 1kb
0 4 8
0 1.5 3
0 15 30
0 20 40
0 1 2 3

**Supplementary Figure 1. Characterization of ChIATAC data in *Drosophila* S2 cells.**

**a** 2D contact matrices of ChI**A**TAC (top triangle) vs. RNAPII ChIA-PET (bottom triangle) at different resolutions in S2 cells. **b** Example views of genome browser tracks for chromatin interaction loops and open chromatin peaks from three replicates of ChI**A**TAC data. Tracks of ATAC-seq, RNAPII ChIA-PET (loops and peaks), ChIP-seq of H3K27ac, H3K27me3, and RNA-seq are included as references. The signal intensity scales (y-axis) of peaks (maximum of reads pileup) and loops (maximum of PET counts) for each track are provided. **c** Spearman correlation of the read coverage with 10-kb bin size between replicates of ChI**A**TAC. **d** Top, Venn diagrams of peak overlap between ChI**A**TAC replicates (R1, n=12,721; R2, n=11,957; R3, n=13,814). Bottom, violin plots of peak intensity for overlapping peaks and replicate-unique peaks. In the box plots, middle line denotes median; box denotes interquartile range (IQR); and whiskers denote 1.5× IQR. **e** Heatmaps of signal intensities for chromatin features in the vicinity (±1 kb) of peaks mapped by ChI**A**TAC (n=23,759): ATAC-seq, and chromatin immunoprecipitation of RNAPII and the histone epigenetic marks H3K27ac and H3K27me3 in S2 cells. Source data are provided as a Source Data file.

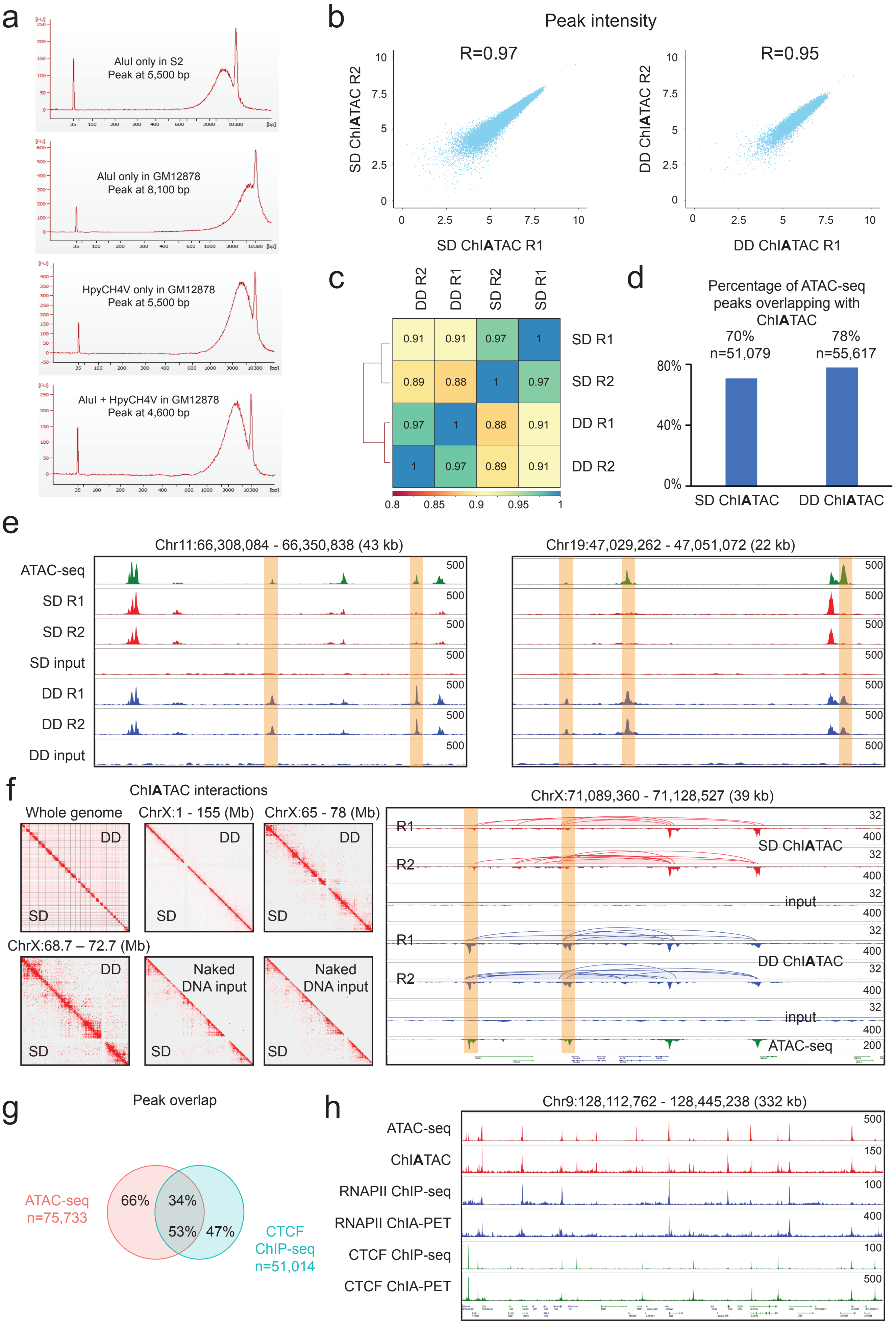
a
AluI only in S2
Peak at 5,500 bp
AluI only in GM12878
Peak at 8,100 bp
HpyCH4V only in GM12878
Peak at 5,500 bp
AluI + HpyCH4V in GM12878
Peak at 4,600 bp
b
Peak intensity
R=0.97
R=0.95
SD ChIATAC R2
SD ChIATAC R1
DD ChIATAC R2
DD ChIATAC R1
c
DD R2
DD R1
SD R2
SD R1
0.91 0.91 0.97 1
0.89 0.88 1 0.97
0.97 1 0.88 0.91
1 0.97 0.89 0.91
0.8 0.85 0.9 0.95 1
d
Percentage of ATAC-seq peaks overlapping with ChIATAC
70%
n=51,079
78%
n=55,617
80%
40%
0%
SD ChIATAC
DD ChIATAC
e
Chr11:66,308,084 - 66,350,838 (43 kb)
Chr19:47,029,262 - 47,051,072 (22 kb)
ATAC-seq
SD R1
SD R2
SD input
DD R1
DD R2
DD input
500
f
ChIATAC interactions
Whole genome
ChrX:1 - 155 (Mb)
ChrX:65 - 78 (Mb)
ChrX:68.7 – 72.7 (Mb)
DD
SD
Naked DNA input
ChrX:71,089,360 - 71,128,527 (39 kb)
R1
R2
SD ChIATAC
input
DD ChIATAC
ATAC-seq
32
400
200
g
Peak overlap
ATAC-seq
n=75,733
66%
34%
53%
47%
CTCF
ChIP-seq
n=51,014
h
Chr9:128,112,762 - 128,445,238 (332 kb)
ATAC-seq
ChIATAC
RNAPII ChIP-seq
RNAPII ChIA-PET
CTCF ChIP-seq
CTCF ChIA-PET
500
150
100
400

**Supplementary Figure 2. Optimization of ChIATAC protocol and characterization of ChIATAC data in human GM12878 cells.**

**a** Size distribution of DNA fragments after restriction enzyme digestion using AluI or HpyCH4V (single digestion, SD) or AluI+HpyCH4V (double digestion, DD). The chromatin samples prepared by SD and DD were used for ChI**A**TAC analysis. **b** Scatter plots of peak intensity between replicates of SD ChI**A**TAC and DD ChI**A**TAC data. The R-value is Spearman's correlation coefficient. **c** Spearman correlation of read coverage with 10-kb bin size between replicates of SD and DD ChI**A**TAC data. **d** Bar plot showing the percentage of peaks in SD and DD ChI**A**TAC data overlapping with ATAC-seq data (n=75,753). **e** Example view of genome browser tracks of replicates of SD and DD ChI**A**TAC data with their input controls and ATAC-seq. Highlighted are ATAC-seq peaks that were only captured in DD ChI**A**TAC data. The signal intensity scales (y-axis) of peaks (maximum of reads pileup) for each track are provided. **f** Left, 2D contact matrices of SD and DD ChI**A**TAC data and their input controls at various resolutions. Right, example view of genome browser tracks of replicates of SD and DD ChI**A**TAC data with their input controls and ATAC-seq data. The signal intensity scales (y-axis) of peaks (maximum of reads pileup) and loops (maximum of PET counts) for each track are provided. **g** Venn diagram showing the peak overlap between ATAC-seq (n=75,733) and CTCF ChIP-seq (n=51,014). **h** Example view of genome browser tracks of peaks in ATAC-seq, ChI**A**TAC, RNAPII ChIP-seq and ChIA-PET, and CTCF ChIP-seq and ChIA-PET data. The signal intensity scales (y-axis) of peaks (maximum of reads pileup) for each track are provided. Source data are provided as a Source Data file.

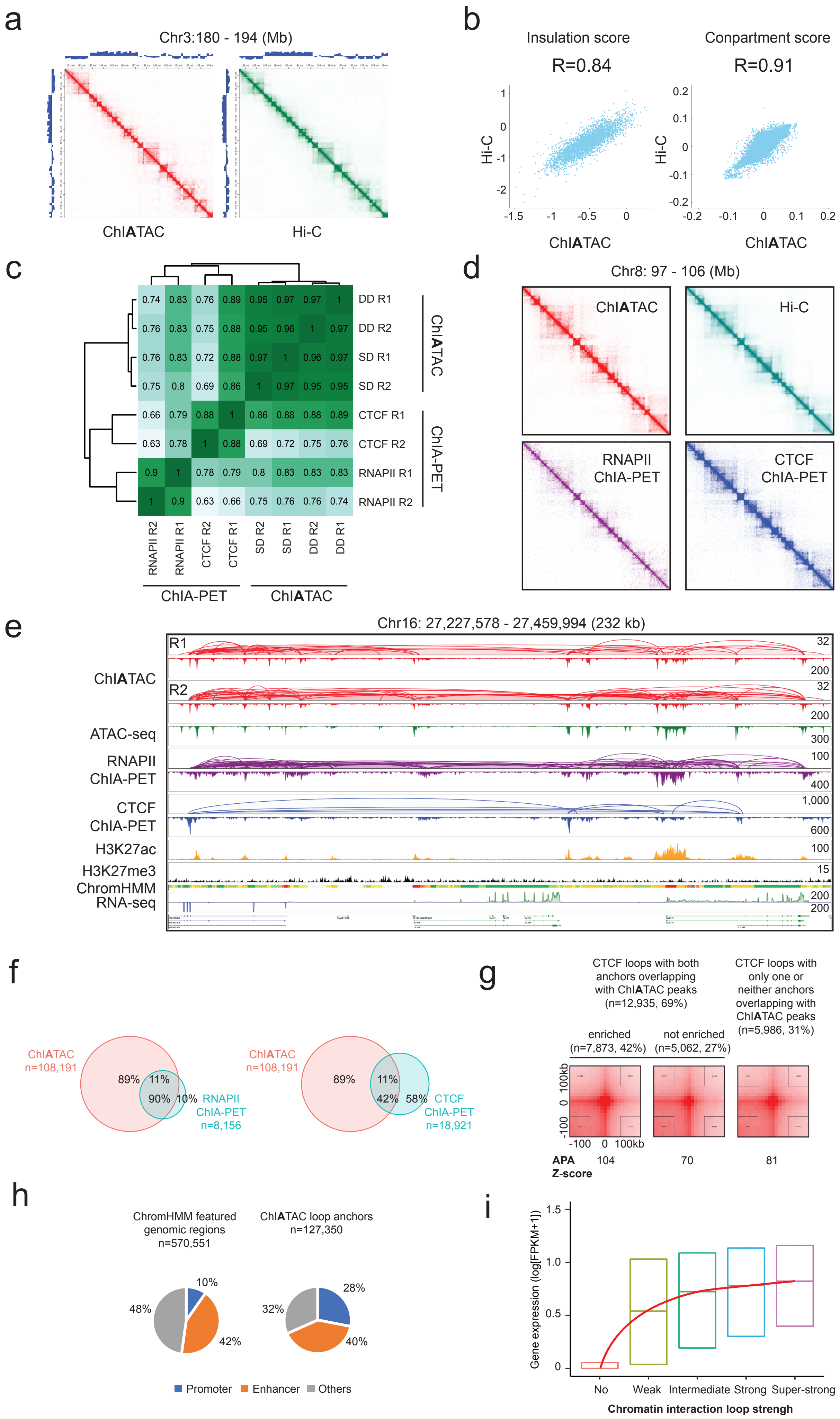
a
Chr3:180 - 194 (Mb)
ChIATAC
Hi-C
b
Insulation score
R=0.84
Hi-C
ChIATAC
Conpartment score
R=0.91
Hi-C
ChIATAC
c
DD R1
DD R2
SD R1
SD R2
CTCF R1
CTCF R2
RNAPII R1
RNAPII R2
ChIATAC
ChIA-PET
d
Chr8: 97 - 106 (Mb)
ChIATAC
Hi-C
RNAPII ChIA-PET
CTCF ChIA-PET
e
Chr16: 27,227,578 - 27,459,994 (232 kb)
ChIATAC
R1
R2
ATAC-seq
RNAPII ChIA-PET
CTCF ChIA-PET
H3K27ac
H3K27me3
ChromHMM
RNA-seq
f
ChIATAC n=108,191
89%
11%
90%
10%
RNAPII ChIA-PET n=8,156
ChIATAC n=108,191
89%
11%
42%
58%
CTCF ChIA-PET n=18,921
g
CTCF loops with both anchors overlapping with ChIATAC peaks (n=12,935, 69%)
CTCF loops with only one or neither anchors overlapping with ChIATAC peaks (n=5,986, 31%)
enriched (n=7,873, 42%)
not enriched (n=5,062, 27%)
APA Z-score
104
70
81
h
ChromHMM featured genomic regions n=570,551
ChIATAC loop anchors n=127,350
10%
42%
48%
28%
40%
32%
Promoter
Enhancer
Others
i
Gene expression (log[FPKM+1])
No
Weak
Intermediate
Strong
Super-strong
Chromatin interaction loop strengh

**Supplementary Figure 3. Additional characterization of ChIATAC data in human GM12878 cells.**

**a** Example view of 2D contact matrices of ChI**A**TAC and Hi-C data along with compartment (eigenvector) track. **b** Scatter plot of insulation score at TAD boundaries (bin size, 25 kb) and compartment score (bin size, 200 kb) in ChI**A**TAC and Hi-C data. The R-value is Spearman's correlation coefficient. **c** Genome-wide reproducibility assessment of chromatin interaction data from replicates of ChI**A**TAC, ChIA-PET of RNAPII, and CTCF data using HiCRep (bin size, 25 kb). **d** Example view of 2D contact matrices (bin size, 25 kb) of ChI**A**TAC, RNAPII ChIA-PET, CTCF ChIA-PET, and Hi-C data derived from human GM12878 cells. **e** Example of genome browser tracks of DD ChI**A**TAC (loop and peak), ATAC-seq (peak), RNAPII ChIA-PET (loop and peak), CTCF ChIA-PET (loop and peak), ChIP-seq (peak) of H3K27ac and H3K27me3, ChromHMM, and RNA-seq data. The signal intensity scales (y-axis) of peaks (maximum of reads pileup) and loops (maximum of PET counts) for each track are provided. **f** Venn diagram showing the overlapping loops between ChI**A**TAC (n=108,191) and RNAPII ChIA-PET (PET≥15, n=8,156) or CTCF ChIA-PET (PET≥15, n=18,921). **g** APA plots of three categories of CTCF ChIA-PET loops: Left, CTCF loops with both anchors overlapping with ChI**A**TAC peaks with enrichment (n=7,873, 42%); Middle, CTCF loops with both anchors overlapping with ChI**A**TAC peaks without enrichment (n=5,062, 27%); Right, CTCF loops with only one or neither anchor overlapping with ChI**A**TAC peaks (n=5,986, 31%). **h** Categorization of ChromHMM annotated genomic regions in GM12878 cells. Left, ChromHMM annotated genomic regions (n=570,511). Right, ChI**A**TAC loop anchor sites (n=122,756). **i** Correlation between loop strength and loop-targeted gene expression in FPKM (fragments per kilobase exon per million mapped reads). For 10,767 genes with loops connected to their promoters (TSS±2.5 kb), we ranked them according to contact frequency (numbers of PETs): weak (n=2,986, PETs=3-8), intermediate (n=2,115, PETs=9-15), strong (n=2,341, PETs=16-28), and super-strong (n=3,325, PESs=29-415). Genes without loops (No, n=11,134) were used as control. The lower, middle, and upper hinges in the boxplot correspond to the first (Q1), median and third quartiles (Q3). A trend line connecting the medians of the gene expression of each group is shown. Source data are provided as a Source Data file.

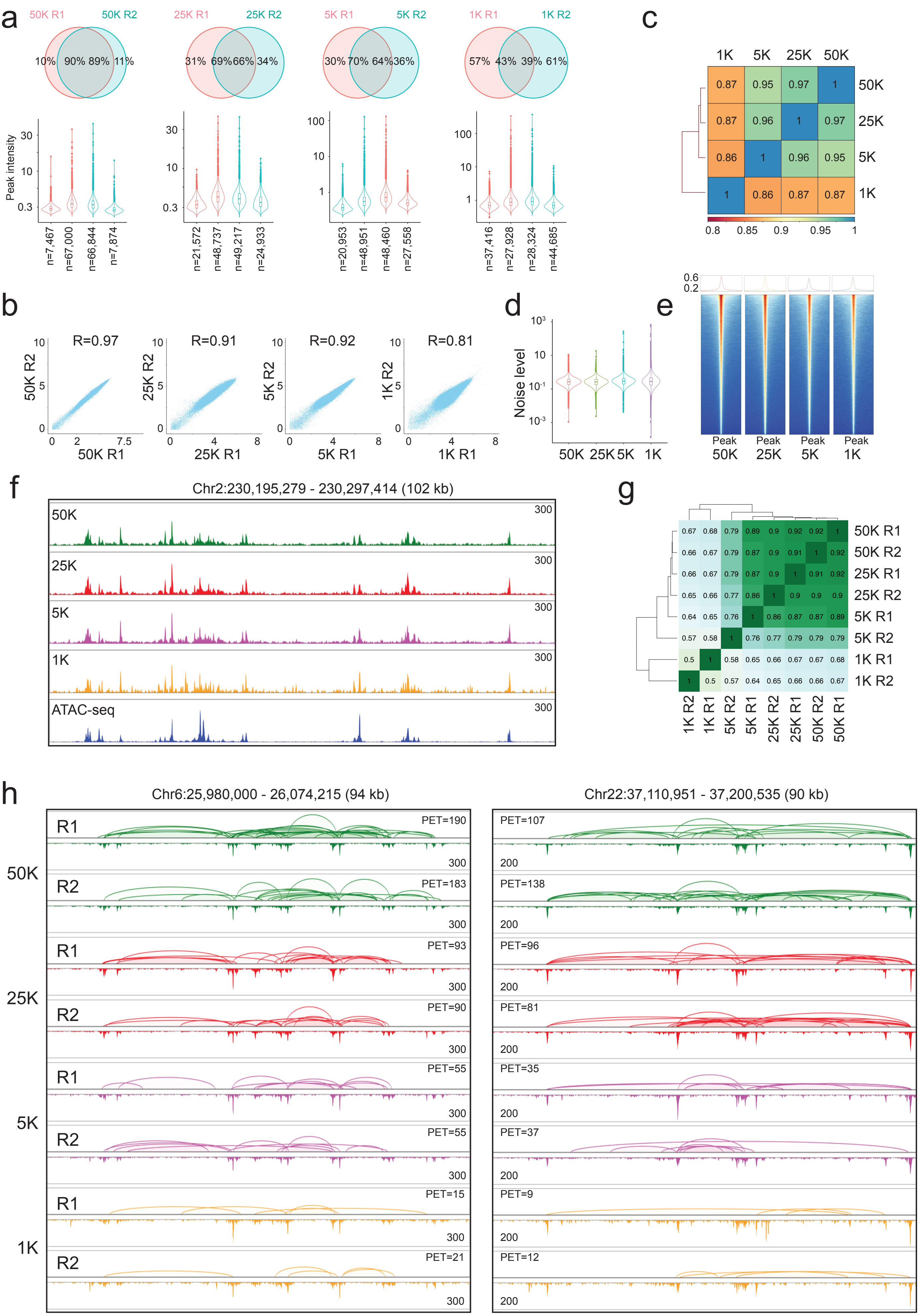

a
50K R1
50K R2
10%
90%
89%
11%
25K R1
25K R2
31%
69%
66%
34%
5K R1
5K R2
30%
70%
64%
36%
1K R1
1K R2
57%
43%
39%
61%
Peak intensity
n=7,467
n=67,000
n=66,844
n=7,874
n=21,572
n=48,737
n=49,217
n=24,933
n=20,953
n=48,951
n=48,460
n=27,558
n=37,416
n=27,928
n=28,324
n=44,685
b
R=0.97
R=0.91
R=0.92
R=0.81
50K R2
50K R1
25K R2
25K R1
5K R2
5K R1
1K R2
1K R1
c
1K
5K
25K
50K
0.87 0.95 0.97 1
0.87 0.96 1 0.97
0.86 1 0.96 0.95
1 0.86 0.87 0.87
0.8
0.85
0.9
0.95
1
d
Noise level
50K
25K
5K
1K
e
Peak
50K
25K
5K
1K
f
Chr2:230,195,279 - 230,297,414 (102 kb)
50K
25K
5K
1K
ATAC-seq
300
g
50K R1
50K R2
25K R1
25K R2
5K R1
5K R2
1K R1
1K R2
0.67 0.68 0.79 0.89 0.9 0.92 0.92 1
0.66 0.67 0.79 0.87 0.9 0.91 1 0.92
0.66 0.67 0.79 0.87 0.9 1 0.91 0.92
0.65 0.66 0.77 0.86 1 0.9 0.9 0.9
0.64 0.65 0.76 1 0.86 0.87 0.87 0.89
0.57 0.58 1 0.76 0.77 0.79 0.79 0.79
0.5 1 0.58 0.65 0.66 0.67 0.67 0.68
1 0.5 0.57 0.64 0.65 0.66 0.66 0.67
h
Chr6:25,980,000 - 26,074,215 (94 kb)
Chr22:37,110,951 - 37,200,535 (90 kb)
50K
25K
5K
1K
R1
R2
PET=190
PET=107
PET=183
PET=138
PET=93
PET=96
PET=90
PET=81
PET=55
PET=35
PET=55
PET=37
PET=15
PET=9
PET=21
PET=12
300
200

**Supplementary Figure 4. Input cell number titration analysis.**

**a** Top, Venn diagrams of peak overlap between replicates of ChI**A**TAC data produced from 50,000 down to 1,000 cells. Bottom, violin plots of peak intensity for overlapping peaks and replicate-unique peaks. In the box plot, middle line denotes median; box denotes interquartile range (IQR); and whiskers denote 1.5× IQR. **b** Scatter plots of read coverage between replicates of ChI**A**TAC data produced from 50,000 down to 1,000 cells with bin size in 10 kb. **c** Spearman correlation of the read coverage with bin size in 10 kb between ChI**A**TAC data produced from 50,000 down to 1,000 cells (50K, 25K, 5K, 1K). **d** Violin plot showing the noise-to-signal ratio of ChI**A**TAC data produced from 50,000 down to 1,000 cells. In the box plot, middle line denotes median; box denotes interquartile range (IQR); and whiskers denote 1.5× IQR. **e** Heatmap and average profile plot of open chromatin sites (±2.5 kb genomic regions) captured in ChI**A**TAC data produced from 50,000 down to 1,000 cells. Peaks called from 50,000 cell ChI**A**TAC data were used as a reference and sorted in descending order based on intensity. **f** Example of genome browser tracks of coverage tracks of ChI**A**TAC data produced from 50,000 down to 1,000 cells. Also shown is ATAC-seq data. The signal intensity scales (y-axis) of peaks (maximum of reads pileup) for each track are provided. **g** Genome-wide reproducibility assessment of chromatin interaction data from replicates of ChI**A**TAC data produced by using from 50,000 down to 1,000 cells using HiCRep (bin size, 25 kb). **h** Example views of genome browser tracks of replicates of ChI**A**TAC data produced from 50,000 down to 1,000 cells. The total number of interacting PET counts within the genomic region is shown. The signal intensity scales (y-axis) of peaks (maximum of reads pileup) and loops (maximum of PET counts) for each track are provided. Source data are provided as a Source Data file.

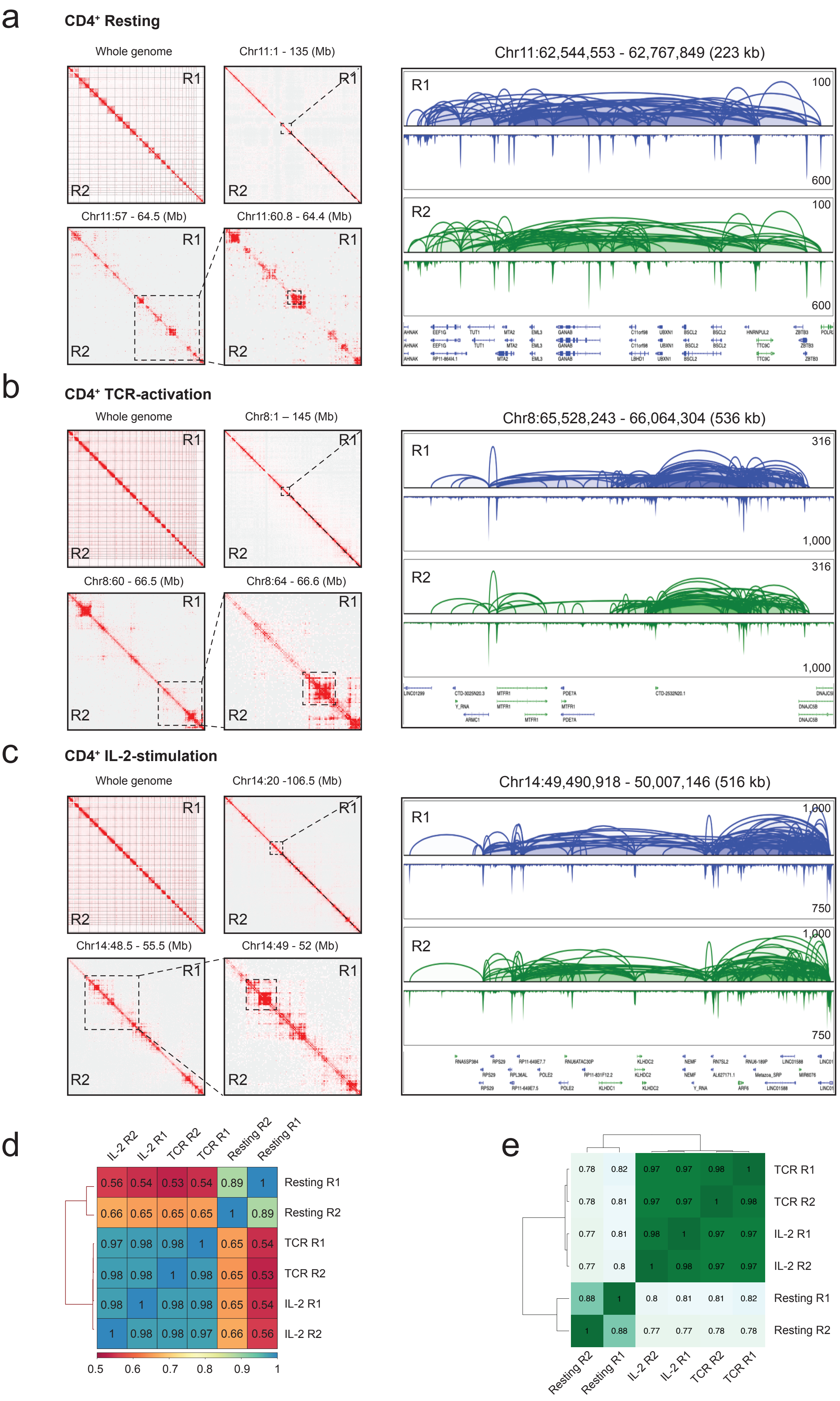
a
CD4⁺ Resting
Whole genome
Chr11:1 - 135 (Mb)
Chr11:57 - 64.5 (Mb)
Chr11:60.8 - 64.4 (Mb)
Chr11:62,544,553 - 62,767,849 (223 kb)
R1
R2
100
600
b
CD4⁺ TCR-activation
Whole genome
Chr8:1 – 145 (Mb)
Chr8:60 - 66.5 (Mb)
Chr8:64 - 66.6 (Mb)
Chr8:65,528,243 - 66,064,304 (536 kb)
316
1,000
c
CD4⁺ IL-2-stimulation
Whole genome
Chr14:20 -106.5 (Mb)
Chr14:48.5 - 55.5 (Mb)
Chr14:49 - 52 (Mb)
Chr14:49,490,918 - 50,007,146 (516 kb)
1,000
750
d
IL-2 R2 IL-2 R1 TCR R2 TCR R1 Resting R2 Resting R1
0.56 0.54 0.53 0.54 0.89 1 Resting R1
0.66 0.65 0.65 0.65 1 0.89 Resting R2
0.97 0.98 0.98 1 0.65 0.54 TCR R1
0.98 0.98 1 0.98 0.65 0.53 TCR R2
0.98 1 0.98 0.98 0.65 0.54 IL-2 R1
1 0.98 0.98 0.97 0.66 0.56 IL-2 R2
0.5 0.6 0.7 0.8 0.9 1
e
0.78 0.82 0.97 0.97 0.98 1 TCR R1
0.78 0.81 0.97 0.97 1 0.98 TCR R2
0.77 0.81 0.98 1 0.97 0.97 IL-2 R1
0.77 0.8 1 0.98 0.97 0.97 IL-2 R2
0.88 1 0.8 0.81 0.81 0.82 Resting R1
1 0.88 0.77 0.77 0.78 0.78 Resting R2
Resting R2 Resting R1 IL-2 R2 IL-2 R1 TCR R2 TCR R1

**Supplementary Figure 5. Quality assessment of ChIATAC data in primary CD4$^+$ T cells.** **a** ChI**A**TAC data from resting CD4$^+$ T cells. Left, 2D contact matrices at various resolutions from replicates of ChI**A**TAC data. Right, example view of genome browser tracks showing chromatin interaction loops and open chromatin accessible peaks from replicates of ChI**A**TAC data. The signal intensity scales (y-axis) of peaks (maximum of reads pileup) and loops (maximum of PET counts) for each track are provided. **b** ChI**A**TAC data from TCR-activated CD4$^+$ T cells. Left, 2D contact matrices at various resolutions from replicates of ChI**A**TAC data. Right, example view of genome browser tracks showing chromatin interaction loops and open chromatin accessible peaks from replicates of ChI**A**TAC data. The signal intensity scales (y-axis) of peaks (maximum of reads pileup) and loops (maximum of PET counts) for each track are provided. **c** ChI**A**TAC data from IL-2-stimulated CD4$^+$ T cells. Left, 2D contact matrices at various resolutions from replicates of ChI**A**TAC data. Right, example view of genome browser tracks showing chromatin interaction loops and open chromatin accessible peaks from replicates of ChI**A**TAC data. The signal intensity scales (y-axis) of peaks (maximum of reads pileup) and loops (maximum of PET counts) for each track are provided. **d** Spearman correlation of read coverage between replicates (R1 and R2) of ChI**A**TAC data at 3 states of CD4$^+$ T cells: resting, TCR-activation, and IL-2-stimulation. **e** Genome-wide reproducibility assessment of chromatin interaction data of replicates of ChI**A**TAC data at 3 states of CD4$^+$ T cells using HiCRep (bin size=10 kb). The correlation coefficients between the corresponding rows and columns are shown.

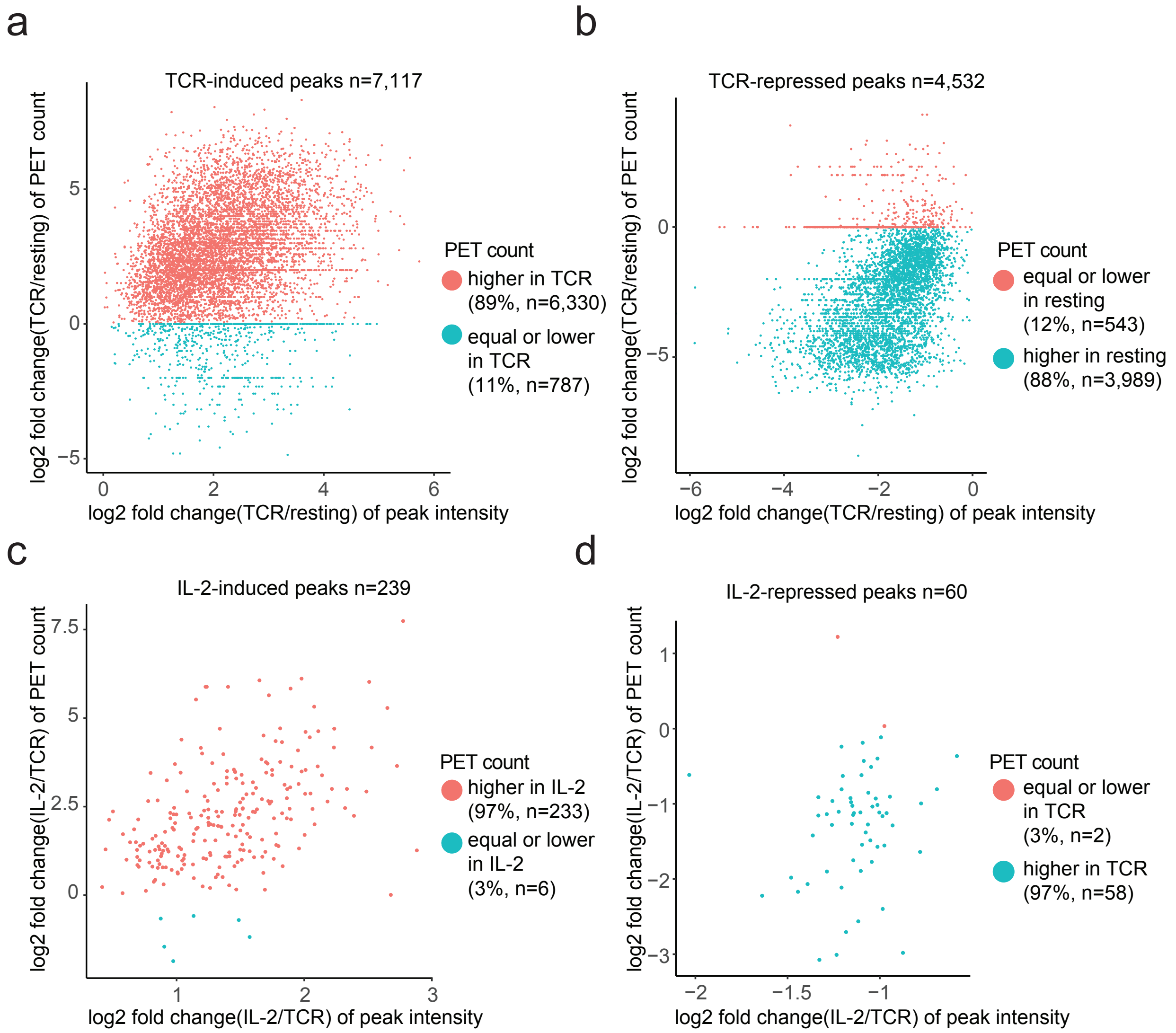
a
TCR-induced peaks n=7,117
log2 fold change(TCR/resting) of PET count
log2 fold change(TCR/resting) of peak intensity
PET count
higher in TCR (89%, n=6,330)
equal or lower in TCR (11%, n=787)
b
TCR-repressed peaks n=4,532
log2 fold change(TCR/resting) of PET count
log2 fold change(TCR/resting) of peak intensity
PET count
equal or lower in resting (12%, n=543)
higher in resting (88%, n=3,989)
c
IL-2-induced peaks n=239
log2 fold change(IL-2/TCR) of PET count
log2 fold change(IL-2/TCR) of peak intensity
PET count
higher in IL-2 (97%, n=233)
equal or lower in IL-2 (3%, n=6)
d
IL-2-repressed peaks n=60
log2 fold change(IL-2/TCR) of PET count
log2 fold change(IL-2/TCR) of peak intensity
PET count
equal or lower in TCR (3%, n=2)
higher in TCR (97%, n=58)

**Supplementary figure 6. Scatter plots of correlation between chromatin accessibility and chromatin connectivity in primary CD4$^+$ T cells.**

**a** TCR-activation induced open chromatin peaks (n=7,117) and associated chromatin loops. **b** TCR-activation repressed open chromatin peaks (n=4,532) and associated chromatin loops. **c** IL-2 induced open chromatin peaks (n=239) and associated chromatin loops. **d** IL-2 repressed open chromatin peaks (n=60) and associated chromatin loops. Y-axis: fold change in log2 scale of PET counts in TCR/resting cells or IL-2/TCR stimulated cells; X-axis: fold change in log2 scale of peak intensity in TCR/resting cells or IL-2/TCR stimulated cells. Source data are provided as a Source Data file.

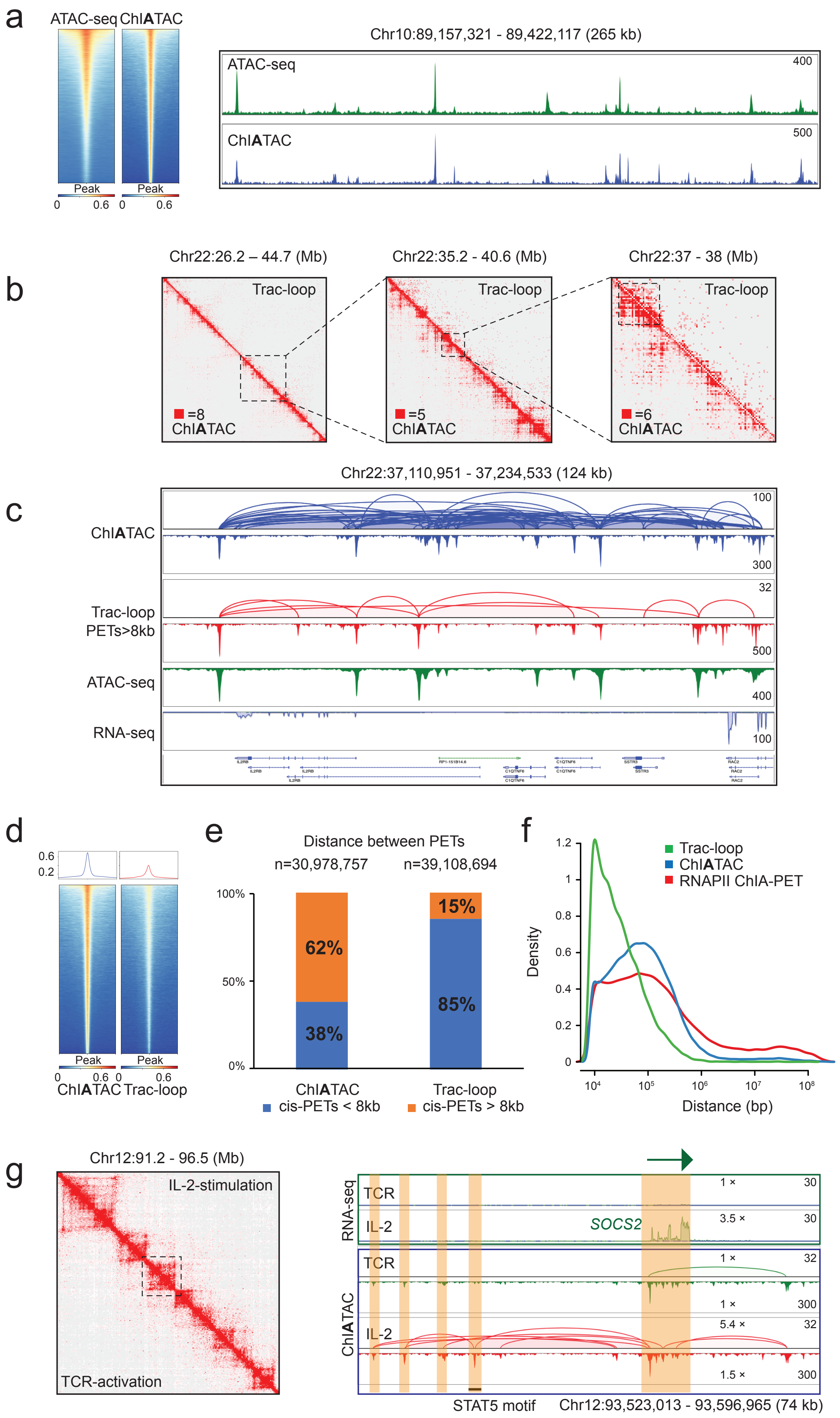

a
ATAC-seq
ChIATAC
Peak
0
0.6
Chr10:89,157,321 - 89,422,117 (265 kb)
400
500
b
Chr22:26.2 – 44.7 (Mb)
Chr22:35.2 - 40.6 (Mb)
Chr22:37 - 38 (Mb)
Trac-loop
=8
=5
=6
ChIATAC
c
Chr22:37,110,951 - 37,234,533 (124 kb)
ChIATAC
Trac-loop
PETs>8kb
ATAC-seq
RNA-seq
100
300
32
500
400
d
0.6
0.2
Peak
ChIATAC
Trac-loop
e
Distance between PETs
n=30,978,757
n=39,108,694
100%
50%
0%
62%
38%
15%
85%
ChIATAC
Trac-loop
cis-PETs < 8kb
cis-PETs > 8kb
f
Trac-loop
ChIATAC
RNAPII ChIA-PET
Density
Distance (bp)
g
Chr12:91.2 - 96.5 (Mb)
IL-2-stimulation
TCR-activation
RNA-seq
TCR
IL-2
SOCS2
1 ×
3.5 ×
30
ChIATAC
5.4 ×
1.5 ×
STAT5 motif
Chr12:93,523,013 - 93,596,965 (74 kb)

**Supplementary Figure 7. Comparison between ChIATAC and Trac-loop data in primary CD4$^+$ T cells.**

**a** Comparison between ATAC-seq and ChI**A**TAC peaks. Left, heatmap signals of open chromatin sites (±2.5 kb genomic regions) measured by ChI**A**TAC data (n=81,883), and the corresponding signals in ATAC-seq data. Signals are normalized. Right, an example view of genome browser tracks of ATAC-seq and ChI**A**TAC identified peaks. The signal intensity scales (y-axis) of peaks (maximum of reads pileup) for each track are provided. **B** 2D contact matrices of ChI**A**TAC and Trac-loop data at various resolutions. **C** Example view of genome browser tracks of ChI**A**TAC, Trac-loop, ATAC-seq, and RNA-seq from TCR-activated CD4$^+$ T cells. The signal intensity scales (y-axis) of peaks (maximum of reads pileup) and loops (maximum of PET counts) for each track are provided. **D** Heatmap signals and average profile plot of open chromatin sites (±2.5 kb genomic regions) measured by ChI**A**TAC data (n=81,883). ChI**A**TAC data were sorted in descending order based on intensity, and the ChI**A**TAC-identified peak loci were used as the reference for sorting Trac-loop. Signals are normalized. **e** Distance of non-redundant PETs in ChI**A**TAC (n=30,978,757) and Trac-loop (n=39,108,694). **f** Profiling of intra-chromosomal loop spans of ChI**A**TAC (n=221,818), RNAPII ChIA-PET (n=88,148), and Trac-loop with PETs > 8 kb (n=17,912) of TCR-activated CD4$^+$ T cells. **g** Left, 2D contact matrices of ChI**A**TAC data in TCR-activated and IL-2-stimulated cells. Right, example view of genome browser tracks of ChI**A**TAC (peak and loop) and RNA-seq of CD4$^+$ T cells after TCR-activation and subsequent IL-2-stimulation states. The STAT5 binding motif is also shown. Highlighted are regions showing increased chromatin accessibility after IL-2-stimulation. Relative fold change (FC) of chromatin accessibility (highlighted regions), loop PET count (given window), and gene expression from 2 cellular states (TCR-activation and IL-2-stimulation) are provided. Chromatin accessibility: 1 ×, 1.5 ×. Loop count:1 ×, 5.4 ×. Gene expression: 1 ×, 3.5 ×. The signal intensity scales (y-axis) of peaks (maximum of reads pileup) and loops (maximum of PET counts) for each track are provided.

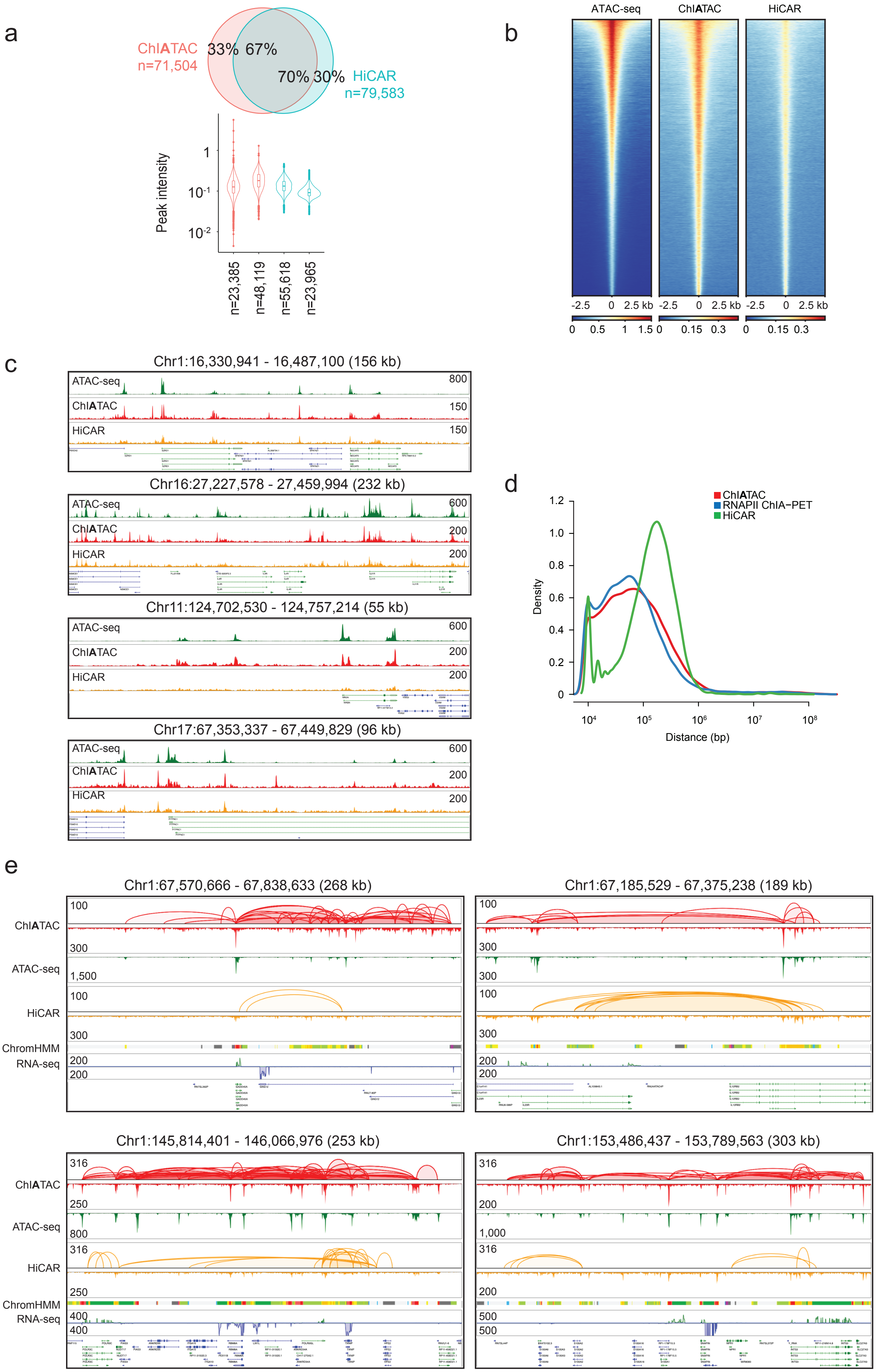

a
ChIATAC
n=71,504
33%
67%
70%
30%
HiCAR
n=79,583
Peak intensity
1
10-1
10-2
n=23,385
n=48,119
n=55,618
n=23,965
b
ATAC-seq
ChIATAC
HiCAR
-2.5
0
2.5 kb
0
0.5
1
1.5
0.15
0.3
c
Chr1:16,330,941 - 16,487,100 (156 kb)
ATAC-seq
800
ChIATAC
150
HiCAR
150
Chr16:27,227,578 - 27,459,994 (232 kb)
ATAC-seq
600
ChIATAC
200
HiCAR
200
Chr11:124,702,530 - 124,757,214 (55 kb)
ATAC-seq
600
ChIATAC
200
HiCAR
200
Chr17:67,353,337 - 67,449,829 (96 kb)
ATAC-seq
600
ChIATAC
200
HiCAR
200
d
ChIATAC
RNAPII ChIA−PET
HiCAR
Density
1.2
1.0
0.8
0.6
0.4
0.2
0
10^4
10^5
10^6
10^7
10^8
Distance (bp)
e
Chr1:67,570,666 - 67,838,633 (268 kb)
Chr1:67,185,529 - 67,375,238 (189 kb)
ChIATAC
ATAC-seq
HiCAR
ChromHMM
RNA-seq
100
300
1,500
200
Chr1:145,814,401 - 146,066,976 (253 kb)
Chr1:153,486,437 - 153,789,563 (303 kb)
316
250
800
400
1,000
500

**Supplementary Figure 8. Comparison between ChIATAC and HICAR data in human GM12878 cells.**

**a** Top, Venn diagrams of peak overlap between ChI**A**TAC (n=71,504) and HiCAR (n=79,583). Bottom, violin plots of peak intensity for overlapping peaks and ChI**A**TAC/HiCAR unique peaks. In the box plot, middle line denotes median; box denotes interquartile range (IQR); and whiskers denote 1.5× IQR. **b** Heatmap signals of open chromatin sites (±2.5 kb genomic regions) measured by ATAC-seq (n=75,753). ATAC-seq data were sorted in descending order based on intensity, and the ATAC-seq loci were used as the reference for sorting ChI**A**TAC and HiCAR peaks. Signals are normalized. **c** Example view of genome browser tracks of ATAC-seq, ChI**A**TAC, and HiCAR from GM12878 cells. The signal intensity scales (y-axis) of peaks (maximum of reads pileup) for each track are provided. **d** Profiling of intra-chromosomal loop spans of ChI**A**TAC (n=108,191), RNAPII ChIA-PET (n=65,697), and HiCAR (n=48,516) of GM12878 cells. **e** Example view of genome browser tracks of ChI**A**TAC, ATAC-seq, HiCAR, ChromHMM (red for active promoter, orange for strong enhancer, yellow for weak/poised enhancer, and green for transcribed region), and RNA-seq in GM12878 cells. The signal intensity scales (y-axis) of peaks (maximum of reads pileup) and loops (maximum of PET counts) for each track are provided. Source data are provided as a Source Data file.

a

ChI**A**TAC data of GM12878 cells (3.26 x $10^7$ non-redundant cis-PETs)

| | Bin-based clustering | | | | Anchor-based clustering | |
|---|---|---|---|---|---|---|
| Resolution | 10 kb (bin size) | | 5 kb (bin size) | | 0.5 kb (peak) | |
| | Loop # | Loop distance | Loop # | Loop distance | Loop # | Loop distance |
| HICCUPS | 8,232 | 170 kb | 5,806 | 110 kb | | |
| Mustache | 687 | 160 kb | 31 | 110 kb | | |
| ChIA-PIPE | | | | | 108,191 | 62.3 kb |

b

ChI**A**TAC data of TCR-activated CD4+ T cells (3.8 x $10^7$ non-redundant cis-PETs)

| | Bin-based clustering | | | | Anchor-based clustering | |
|---|---|---|---|---|---|---|
| Resolution | 10 kb (bin size) | | 5 kb (bin size) | | 0.5 kb (peak) | |
| | Loop # | Loop distance | Loop # | Loop distance | Loop # | Loop distance |
| HICCUPS | 12,359 | 190 kb | 10,996 | 135 kb | | |
| Mustache | 1,015 | 160 kb | 40 | 102.5 kb | | |
| ChIA-PIPE | | | | | 319,916 | 86.1 kb |

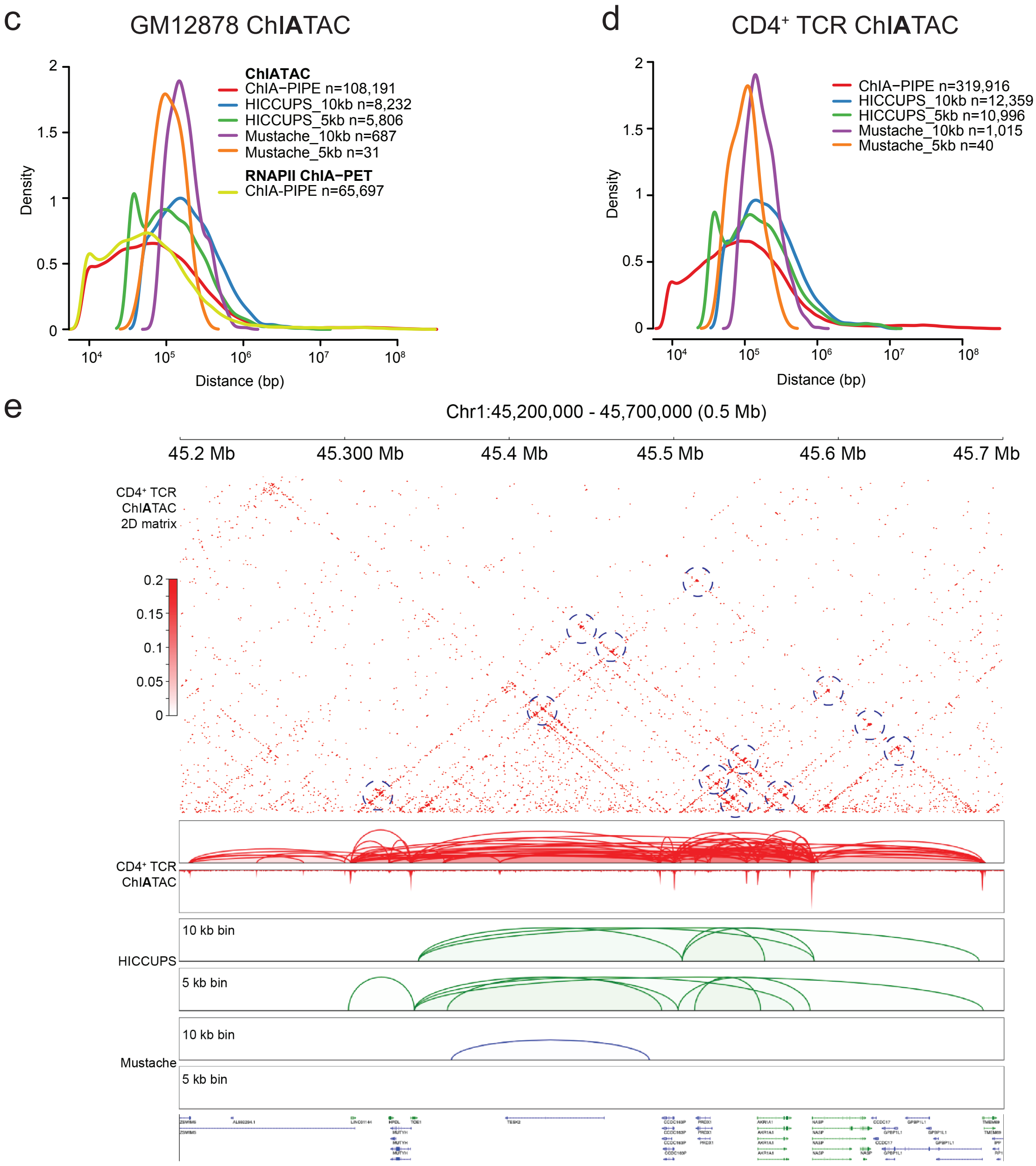

**Supplementary Figure 9. Comparison of chromatin loops called by ChIA-PIPE, HiCCUPS, and Mustache.**

**a** Summary statistics of ChI**A**TAC chromatin loops identified by different methods in GM12878 cells. **b** Summary statistics of ChI**A**TAC chromatin loops identified by different methods in TCR-activated $CD4^+$ T cells. **c** Profiling of intra-chromosomal loop spans of ChI**A**TAC loops called by ChIA-PIPE, HICCUPS with bin size in 10 kb and 5 kb, Mustache with bin size in 10 kb and 5 kb, and RNAPII ChIA-PET loops called by ChIA-PIPE in GM12878 cells. **d** Profiling of intra-chromosomal loop spans of ChI**A**TAC loops called by ChIA-PIPE, HICCUPS with 5 bin size in 10 kb and 5 kb, Mustache with bin size in 10 kb and 5 kb in TCR-activated $CD4^+$ T cells. **e** Example view of 2D contact matrix of ChI**A**TAC along with genome browser tracks of ChI**A**TAC loops called by ChIA-PIPE, HICCUPS with bin size in 10 kb and 5 kb, Mustache with bin size in 10 kb and 5 kb.

**Supplementary Note 1**

**A step-by-step ChIATAC protocol**

***Materials***

2% FA-DPBS solution (see recipe)

2 mM EGS-DPBS solution (see recipe)

Dulbecco's phosphate-buffered saline (DPBS), calcium- and magnesium-free (Gibco, cat. no. 14190-250)

2.5 M glycine (see recipe)

Formaldehyde (FA; 36% v/v; Sigma-Aldrich, cat. no. 47608-250ML-F)

0.1% SDS cell lysis buffer (see recipe)

cOmplete™ Mini EDTA-free Protease Inhibitor Cocktail (PI; Roche, cat. no. 11836170001)

10% Triton X-100, molecular biology grade (Sigma-Aldrich, cat. no. 648464)

10× CutSmart buffer (New England Biolabs)

AluI restriction enzyme (NEB, cat. no. R0137L)

HpyCH4V restriction enzyme (NEB, cat. no. R0620L)

TE buffer, pH 8.0, RNase-free (Thermo Fisher Scientific, cat. no. AM9858)

10 mM dATP solution (NEB, cat. no. N0400S)

Agilent DNA High-Sensitivity Kit (Agilent Technologies, cat. no. 5067-4626)

Bovine serum albumin (BSA, molecular-biology grade, 20 mg/ml; NEB, cat. no. B9000S)

Nuclease-free water (Thermo Fisher Scientific, cat. no. AM9932)

Klenow Fragment (3'→5' exo-) (NEB, cat. no. M0212L)

NEBNext® Quick Ligation Reaction Buffer, 5× (NEB, cat. no. B6058S)

Bridge linker, 2 ng/μl (see Addendum)

T4 DNA ligase (NEB, cat. no. M0202L)

Dynabeads Protein G beads for immunoprecipitation (Thermo Fisher Scientific, cat. no. 10009D)

Buffer EB (Qiagen, cat. no. 19086)

Qubit® dsDNA HS Assay Kit (Thermo Fisher Scientific, cat. no. Q32854)

Nextera XT Index Kit v2 Set A (Illumina, cat. no. FC-131-2001)

Illumina Tagment DNA Enzyme and Buffer Large Kit (Illumina, cat. no. 20034198)

Dynabeads M-280 Streptavidin (Thermo Fisher Scientific, cat. no.11205D)

2x Binding & Wash buffer (see recipe)

iBlock buffer (see recipe)

Sheared genomic DNA mixture (see recipe)

2× SSC/0.5% (w/v) SDS (see recipe)

NEBNext® High-Fidelity 2x PCR Master Mix (NEB, cat. no. M0541S)
AMPure XP beads (60 ml; Beckman, cat. no. A63881)
80% ethanol

DNA LoBind Tubes (1.5 ml, Eppendorf, cat. no. 022431021)
DNA LoBind Tubes (0.5 ml, Eppendorf, cat. no. 022431005)
DynaMag-2 Magnet (magnetic stand; Thermo Fisher Scientific, cat. no. 12321D)
Qubit® Assay tubes (Thermo Fisher Scientific, cat. no. Q32856)
0.2-ml PCR tubes PCR machine (BioRad, C1000 Touch Thermal Cycler)
DNA Clean & Concentrator-5 kit (Zymo Research, cat. no. D4014)
RM-2M Intelli-Mixer, Medium (ELMI, cat. no. IMIX-02)

***Buffers and Reagents:***
FA-DPBS solution, 2%
Add 2941 µl of 36% formaldehyde Sigma-Aldrich, cat. no. 47608-250ML-F) to 50 ml DPBS (Gibco, cat. no. 14190-250).
*Prepare immediately before use.*

EGS-DPBS solution, 2 mM
Take EGS [ethylene glycol bis (succinimidyl succinate] out of the 4 °C refrigerator and equilibrate to room temperature for at least 1 h. Dissolve 45.63 mg of EGS in 250 µl of DMSO with vortexing. Add the EGS/DMSO solution to 50 ml pre-warmed (37 °C) DPBS.
*Prepare immediately before use.*

Glycine solution, 2.5 M
Add 27.89 g of glycine to 80 ml of dd$H_2O$ and mix well until the solution is clear. Bring the final solution volume to 100 ml.
*Store at room temperature for several months.*

0.1% SDS cell lysis buffer
50 mM HEPES-KOH pH 7.5 (Fisher Scientific, cat. no. BP299-1)
150 mM NaCl
1 mM EDTA (Thermo Fisher Scientific, cat. no. 9261)
1% (w/v) Triton X-100 (Sigma-Aldrich, cat. no. 648464)
0.1% (w/v) sodium deoxycholate
0.1% (w/v) sodium dodecyl sulfate (SDS) solution (Thermo Fisher Scientific, cat. no. AM9822)

*Store at 4 °C for several months.*

ATAC wash buffer
10 mM Tris-HCl pH 7.4
10 mM NaCl
3 mM $MgCl_2$
0.1% Tween-20
*Make fresh and keep on ice.*

iBlock buffer
Dissolve 2 g of iBlock Protein-Based Blocking Reagent (Thermo Fisher Scientific, cat. no. T2015) in 90 ml of $ddH_2O$ in a 65 °C water bath, add 5 ml of 10% (w/v) SDS, and bring volume to 100 ml with $ddH_2O$.
*Stored at room temperature for several months.*

Sheared blocking genomic DNA
The sheared genomic DNA can be prepared from any species. Usually, genomic DNA is sheared to an approximate size range of 200-1000 bp. Measure the sheared DNA concentration with Qubit assay. Use 500 ng of sheared DNA for each reaction.
*Stored at -20 °C for several months.*

Binding & Wash buffer, 1×
5 mM Tris-HCl pH 7.5 (Thermo Fisher Scientific, cat. no. 15567027)
0.5 mM EDTA (Thermo Fisher Scientific, cat. no. 9261)
1 M NaCl
*Store at room temperature for several months.*

Binding & Wash buffer, 2×
10 mM Tris-HCl pH 7.5 (Thermo Fisher Scientific, cat. no. 15567027)
1 mM EDTA (Thermo Fisher Scientific, cat. no. 9261)
2 M NaCl
*Store at room temperature for several months.*

2× SSC/0.5% (w/v) SDS
Add 85 ml of $ddH_2O$ to a 100-ml beaker first, and then add sequentially 10 ml of 20× SSC and 5 ml of SDS (10% (wt/vol)) and mix them well.
*Store at room temperature for several months.*

***Dual crosslinking of cells***

1. Freshly prepare the Formaldehyde (FA) - DPBS and EGS - DPBS solution and keep the EGS-DPBS solution in the 37 °C incubator to facilitate dissolving of the EGS.
2. Spin down the cells 2,500 × g, room temperature (RT) for 10 min, and remove all supernatant.
3. Resuspend the cells in 1 ml of 2% FA-DPBS solution and incubate for 20 min at room temperature with agitation.
4. Add 87 µl 2.5 M glycine to the tube.
5. Incubate for 10 min at room temperature with agitation to quench the excess FA.
6. Centrifuge at 2,500 × g, RT for 10 min. Remove supernatant and wash the cells with 1 ml DPBS.

   *Done with FA crosslinking.*
7. Resuspend the cells in 1 ml of EGS - DPBS solution and incubate for 45 min at room temperature with agitation.
8. Add 87 µl 2.5 M glycine to the tube.
9. Incubate for 10 min at room temperature with agitation to quench the excess EGS.
10. Centrifuge at 2,500 × g, RT for 10 min. Remove supernatant and wash the cells with 1 ml DPBS.

    *Done with double crosslinking.*
11. Aliquot cells in 50,000 cells in 0.5 ml low binding tube and use directly for ChIATAC or put in -80 °C freezer for long-term storage.

***Cell lysis, nuclei permeabilization, and in situ restriction digestion***

1. Take out a 50,000 dual crosslinked cell pellet from -80 °C freezer, leave it on ice for 20 min to thaw the cells, centrifuge the tube at 2,500 × *g*, 4°C and carefully discard the supernatant.
2. Resuspend cells with 100 µl 0.1% SDS cell lysis buffer (+ Proteinase inhibitor (PI)) and keep on ice for 1 h.
3. Centrifuge the tube at 2,500 × *g*, 4 °C for 5 min and then carefully discard supernatant (leave ~ 5 µl).
4. Re-suspend the cell pellet with 10 µl 0.1% SDS solution (+PI).

   *The condition for nuclei permeabilization (incubation time and SDS concentration) has to be optimized for different cells used. For GM12878, incubate cells with 0.1% SDS + PI for 1.5 h at RT with agitation (UU25).*
5. Incubate the tube at RT for 1.5 h.
6. Add 2.5 µl 20% Triton X-100 solution to the tube.

7. Incubate the tube at 37 °C for 15 min with agitation to quench SDS (UU25).
8. Add 5 µl 10× NEB CutSmart buffer and 1 µl AluI, 1 µl HpyCH4V restriction enzymes, and 25.5 µl $ddH_2O$ to the tube from step 7.
   *Final volume 50 µl*
9. Incubate the tube at a 37 °C incubator for at least 2 h or overnight with agitation (UU25).

***A-tailing & in situ Proximity ligation***

10. Set up A-Tailing reaction on ice in the order as shown below:
    50 µl Restriction digested sample
    1 µl BSA (2 mg/ml)
    0.6 µl 1 mM dATP
    1 µl Klenow Large fragment (3'→5' exo-)
11. Mix well, incubate the tube at 37 °C for 1 h with agitation (UU25).
12. Incubate the tube at 65 °C for 20 min without agitation.
13. Leave the sample tube at RT, add proximity ligation reagents in the order as shown below:
    52.6 µl A-tailed sample (from step 12)
    23.4 µl Nuclease-free water
    3 µl Bridge linker (2 ng/µl)
    1 µl T4 DNA ligase
    20 µl NEB 5× quick ligation buffer

    *Final volume 100 µl, add quick ligation buffer at last*
14. Mix well and incubate the tube at RT for at least 4 h or 16 °C incubator for overnight ligation with agitation (UU25).

***In situ tagmentation***

15. Centrifuge the tube at 2,500 × *g*, 4°C, for 5 min, carefully remove the supernatant (leave ~10 µl).
16. Wash with 100 µl ATAC washing buffer once.
17. Centrifuge the tube at 2,500 × *g*, 4 °C, carefully remove the supernatant (leave ~5 µl).
18. Resuspend cells in 14 µl DPBS.
19. Add 25 µl 2× TDB buffer, and 6 µl TDE enzyme.
    *Final 50 µl volume*
20. Incubate at 37 °C with agitation for 1 h.

21. Purify the DNA by using Zymo DNA Clean and Concentrator-5 Kit, elute with 50 μl EB buffer.

***Biotin enrichment***

22. Equilibrate M-280 streptavidin dynabeads to RT for 30 min, fully resuspend and transfer 30 μl beads into a new tube.
23. Place tube on magnetic stand, discard supernatant and wash beads with 150 μl 2× Binding & Washing buffer twice.
24. Resuspend beads in 100 μl iBlock Buffer, mix and incubate at RT for 45 min with agitation.
25. Discard iBlock buffer with the help of magnetic stand, wash beads with 200 μl of 1× Binding and Washing buffer twice.
26. Discard wash buffer, then add the 100 μl blocking mixture (500 ng genomic DNA in 50 μl nuclease-free water + 50 μl 2× Binding and Washing buffer) to reduce non-specific DNA with no biotin labelling binding to the M-280 beads.
    *The genomic DNA can be prepared from any species.*
27. Mix well with the iBlocked beads, then incubate for 30 min with agitation at RT.
28. Discard supernatant, wash beads with 200 μl of 1× Binding and Washing buffer twice.
29. Add purified DNA (50 μl) to the tube, add 50 μl 2× Binding and Washing buffer, mix well, incubate at RT for 45 min with agitation.
30. Short spin the tube, place tube on magnetic stand, discard supernatant, wash beads with 500 μl 0.5% SDS/ 2× SSC buffer for five times.
31. Wash the M-280 beads with 500 μl 1× Binding and Washing buffer twice.
    *Keep M280 beads on the magnetic stand all times during wash.*
32. Discard supernatant, resuspend beads in 45 μl EB buffer.

***Library construction***

33. Prepare the Illumina library PCR reaction as below:
    15 μl DNA Library-coated beads
    25 μl NEBNext® High-Fidelity 2× PCR Master Mix
    5 μl Index Primer 1 (i5)
    5 μl Index Primer 2 (i7)
    Total 50 μl
34. Amplify in situ ChIA-PET libraries using the follow PCR conditions:
    Initial denaturation at 72 °C for 3 min, then at 98 °C 30 s
    9 - 13 cycles of 10 s 98 °C → 30 s 63 °C → 40 s 72 °C

Final extension at 72 °C for 5 min

Hold at 4 °C.

35. Transfer 50 µl PCR product supernatant from the reaction tube to a new tube with the help of magnetic stand.
36. Purify the DNA by using 1x (50 µl) AMPure XP beads (Equilibrate to RT for 30 min before using).
37. Perform PCR reactions using the rest DNA Library-coated beads as mentioned above.
38. Combine all PCR products (3 times), remove primer dimers and short fragments lacking contact information by using 0.8x AMPure XP beads.
39. Measure the concentration of the PCR product from step 38 with Qubit and check the DNA profile with a Bioanalyzer2100 HS DNA chip.

    *A typical library profile ranging from 200 bp – 1000 bp is shown in Figure1.*

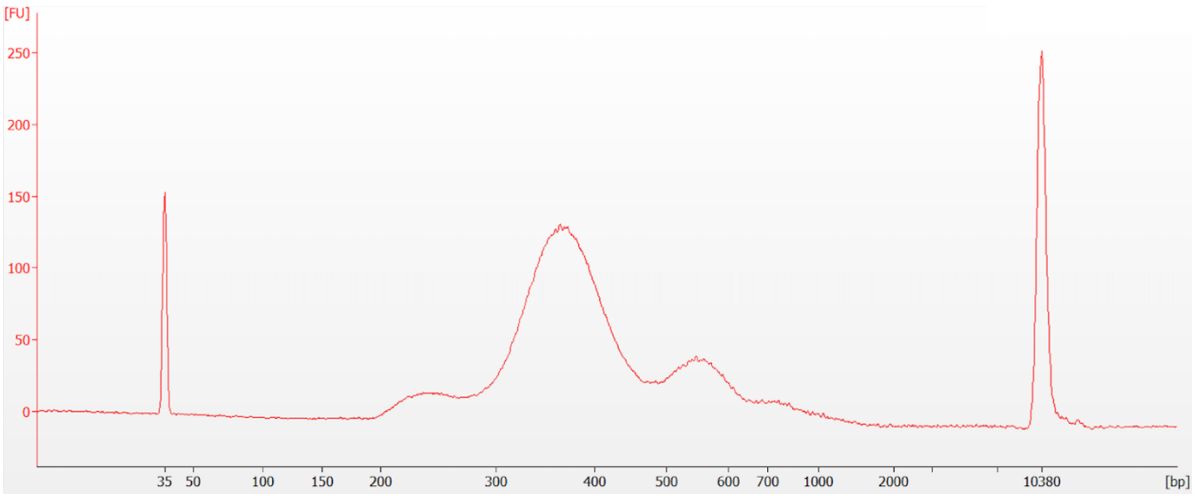


**Supplementary Figure10.** Final ChIATAC library profile.

***Data processing and Visualization***

Adopted from Wang, P., Feng, Y., Zhu, K., Chai, H., Chang, Y., Yang, X., Liu, X., Shen, C., Gaga, E., Lee, B., Kim, M., Ruan, X., & Ruan, Y. (2021). In situ chromatin interaction analysis using paired-end tag sequencing. Current Protocols, 1, e174.

***Materials***

ChIA-PIPE package (https://github.com/TheJacksonLaboratory/ChIA-PIPE.git),v1.0

Sequencing results (steps below are shown for a small test dataset, available at Zenodo, doi: 10.5281/zenodo.4706038)

***ChIA-PIPE***

1. Make a test directory in the home directory of the high-performance computing environment.
   $ mkdir -p testing_chia_pipe
   $ cd testing_chia_pipe
2. Clone the ChIA-PIPE package (v1.0) from the github repository. If git is installed, type:
   $ git clone
   https://github.com/TheJacksonLaboratory/ChIAPIPE.git
   If git is not installed, download ChIA-PIPE directly: Wang et al.
   $ wget
   git@github.com:TheJacksonLaboratory/chia_pipe.zip
3. Install the dependencies for ChIA-PIPE:
   $ dep_dir="dep_dir"
   $ bash local_install_chia_pipe_dependencies.sh -i
   ${dep_dir}
4. Download test data from Zenodo: https://zenodo.org/
   record/4706038#.YIAx2R0pCHs.
   $ mkdir -p fastq
   $ cp LDK0004-ds_*.fastq.gz fastq
5. Review the config file in chia_pipe-master/example_config_file.sh and ensure that bin_dir is specified according to the directory where ChIA-PIPE has been installed.
6. Launch ChIA-PIPE.
   $ qsub -F "--conf
   chia_pipe-master/example_config_file.sh"
   chia_pipe-master/0.chia_pipe_hpc.pbs
7. Wait for the pipeline to finish running. Note that this is expected to take 5-10 h depending on the user's computing environment. After the run, there should be a 4.LDK0004-ds.extract_summary_stats.o and a LDK0004-ds.final_stats.tsv file in the /LDK0004-ds/ directory.
8. Transfer the 7 key processed files in the /LDK0004-ds/ directory from HPC environment to the local desktop for downstream visualization and analyses.
   **a.** LDK0004-ds.final_stats.tsv (As shown in Table 1)
      A summary statistics table including the total read pairs, uniquely mapped read pairs, number of peaks, and number of PET clusters (loops).
   **b.** LDK0004-ds.e500.clusters.cis.gz

A list of intra-chromosomal loops in bedpe format, with 7th columns denoting the number of PETs contributing to a particular loop (7th column also referred to as PET count). In other words, this file can be considered as a table with 7 columns: chrom1, start1, end1, chrom2, start2, end2, PET count.

**c.** LDK0004-ds.e500.clusters.cis.BE3

A subset of LDK0004-ds.e500.clusters.cis.gz with 7th column ≥ 3.

**d.** LDK0004-ds.e500.clusters.trans.gz

A list of inter-chromosomal loops in bedpe format, with 7th column denoting PET count.

**e.** LDK0004-ds.for.BROWSER.sorted.bedgraph

The protein binding coverage file in a standard 4-column bedgraph format.

**f.** LDK0004-ds.no_input_all_peaks.narrowPeak

A list of peaks called by MACS2 in a bed format.

**g.** ChIA-PET_dm3_Kc167_RNAPII_LDK0004-ds_miseq_pairs.hic

A binary file that can be visualized through Juicebox.

9. Visualize intra-chromosomal loops and coverage (As shown in Figure 3).

**a.** Download and install the dockerized version of the BASIC Browser:

https://github.com/TheJacksonLaboratory/basic-browser.

**b.** Follow the github instructions

Upload LDK0004-ds.e500. clusters.cis.BE3 (loops), LDK0004-ds.for.BROWSER.sorted. bedgraph (binding coverage), and LDK0004-ds.no_input_all_peaks.narrowPeak (peaks).

Alternatively, the data can be visualized via the WashU Epigenome browser: https://epigenomegateway.wustl.edu.

10. Visualiz inter- and intra-chromosomal interactions through 2D contact maps (As shown in Figure 2).

**a.** Transfer *.hic file from the high-performance computing environment to the local drive (e.g., Desktop).

**b.** On a web browser, visit https://aidenlab.org/juicebox/.

**c.** Click on “Load Map”, “Local File” and locate the ChIAPET_dm3_Kc167_RNAPII_LDK0004-ds_miseq_pairs.hic file.

*Alternatively, 2D contact maps can be visualized via higlass (https://higlass.io) and the 3D genome browser (http://3dgenome.fsm.northwestern.edu).*

**Supplementary Table 2**. Example QC table(final_stat.tsv) from ChIA-PIPE output.

| | |
|---|---|
| **Library_ID** | **THN0009** |
| Reference_genome | hg38 |
| **Total_PE_reads** | **816,888,402** |
| Linker_detected_in_pair_reads | 773,742,758 |
| PET_with_linker | 288,375,772 |
| Uniquely_mapped_PET | 200,177,382 |
| Non-redundant_PET | 41,428,174 |
| **Redundancy** | **0.79** |
| Self-ligation_PET | 11,806,096 |
| Inter-ligation_PET | 29,622,078 |
| Intra-chr_PET | 19,172,661 |
| Inter-chr_PET | 10,449,417 |
| **ratio_of_intra/inter_PET** | **1.83** |
| Singleton | 24,329,743 |
| Intra-chr_singleton | 15,053,457 |
| Inter-chr_singleton | 9,276,286 |
| PET_cluster | 1,808,220 |
| **ratio_of_intra/inter_cluster** | **2.7** |
| Intra-chr_PET_cluster | 1,319,927 |
| pets_number_2 | 943,509 |
| pets_number_3 | 180,444 |
| pets_number_4 | 71,227 |
| pets_number_5 | 37,751 |
| pets_number_6 | 22,704 |
| pets_number_7 | 14,996 |
| pets_number_8 | 10,314 |
| pets_number_9 | 7,263 |
| pets_number_10 | 5,163 |
| pets_number>10 | 26,556 |
| Inter-chr_PET_cluster | 488,293 |
| pets_number_2 | 395,018 |
| pets_number_3 | 51,930 |
| pets_number_4 | 18,127 |
| pets_number_5 | 9,314 |
| pets_number_6 | 5,474 |
| pets_number_7 | 3,304 |
| pets_number_8 | 2,017 |
| pets_number_9 | 1,228 |
| pets_number_10 | 761 |
| pets_number>10 | 1,120 |

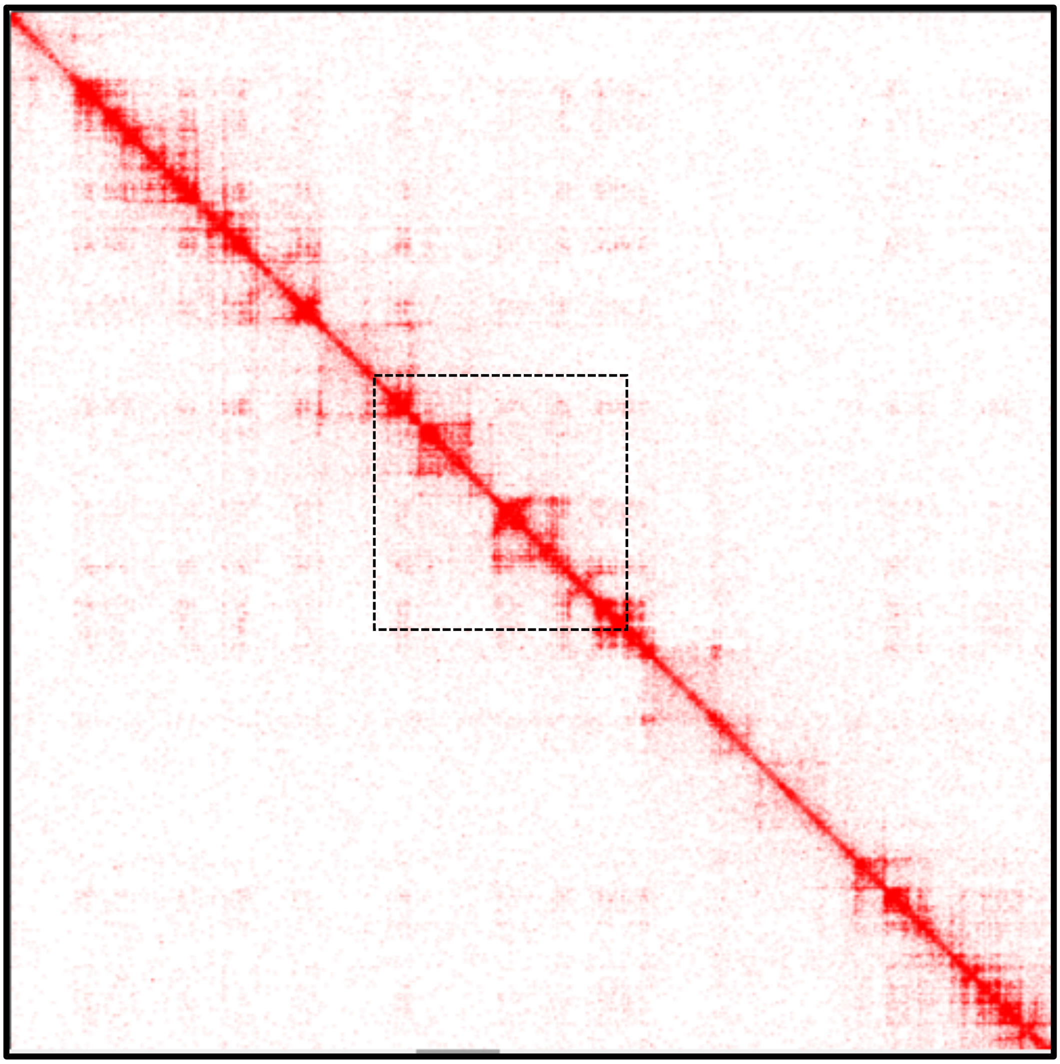

**Supplementary Figure 11.** 2D contact map of ChIATAC data (Chr4: 74.5 Mb – 89 Mb).

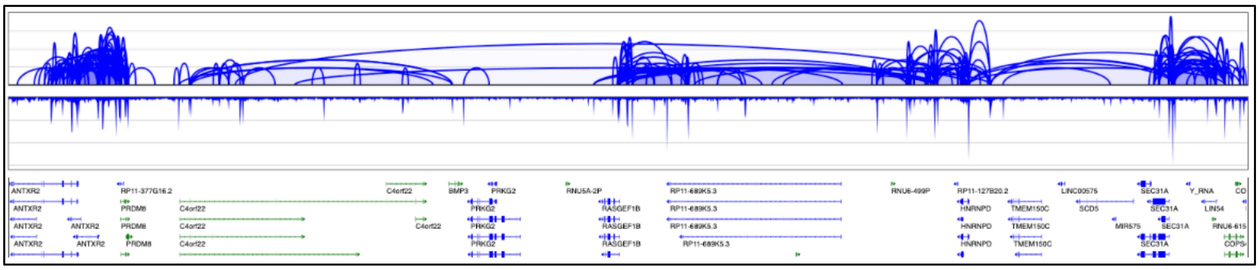


**Supplementary Figure 12.** Zoomed in BASIC browser view of ChIATAC data (79.6 Mb – 82.9 Mb).

**Addendum**

***Bridge linker preparation***

Adopted from Wang, P., Feng, Y., Zhu, K., Chai, H., Chang, Y., Yang, X., Liu, X., Shen, C., Gaga, E., Lee, B., Kim, M., Ruan, X., & Ruan, Y. (2021). In situ chromatin interaction analysis using paired-end tag sequencing. Current Protocols, 1, e174.

***Materials***

DNA oligonucleotides (ordered from IDT or similar, HPLC-purified):
Top oligo: 5’-/5Phos/CG CGA TAT C/iBIOdT/T ATC TGA CT-3’
Bottom oligo: 5’-/5Phos/GT CAG ATA AGA TAT CGC GT-3’
TE buffer, pH 8.0 (Thermo Fisher Scientific, cat. no. AM9849)
NovexTM TBE Gels, 4%-20%, 10 well (Thermo Fisher Scientific, cat. no. EC6225BOX)
25-bp DNA ladder (Thermo Fisher Scientific, cat. no. 10597-011)
TBE buffer (10×), RNase-free (Thermo Fisher Scientific, cat. no. AM9865)
0.2-ml RNase-free PCR tubes (Thermo Fisher Scientific, cat. no. AM12225)
PCR machine (BioRad, C1000 Touch Thermal cycler)
NanoDropTM 8000 Spectrophotometer (Thermo Scientific)
SureLockTM Tandem Midi Gel Tank (Thermo Fisher Scientific, cat. no. STM1001)

1. Order the oligos from IDT (or similar) at 250-nmol scale, HPLC purified in desalted form.
2. Add TE buffer to dissolve the oligos to a final concentration of 100 µM.
3. Vortex to mix well, then place the tubes at 4 °C overnight (without vortexing) to allow oligos to resuspend completely.
4. Prepare five different ratios of top oligo:bottom oligo (1:1, 1.5:1, 2:1, 1:1.5, 1:2) to test for the best annealing efficiency. Ultimately, the selected ratio will be used for large scale annealing of bridge linkers.
Example: for 1.5:1, mix together 7.5 µl of top oligo (100 µM) and 5 µl of bottom oligo (100 µM)
5. Run on a PCR machine using the following program:
95 °C 2 min
Ramp 95 °C to 75 °C (rate: 0.1 °C/s) Hold at 75 °C 2 min
Ramp 75 °C to 65 °C (rate: 0.1 °C/s) Hold at 65 °C 2 min
Ramp 65 °C to 50 °C (Rate of 0.1 °C/s) Hold at 50 °C 2 min
Ramp 50 °C to 37 °C (rate of 0.1 °C/s) Hold at 37 °C 2 min
Ramp 37 °C to 20 °C (rate of 0.1 °C/s) Hold at 20 °C 2 min

Ramp 20 °C to 4 °C (rate of 0.1 °C/s) Hold at 4 °C Indefinitely until collection

6. Measure the concentration of the annealed bridge linkers using NanodropTM 8000 Spectrophotometer (Use the mode for RNA measurement).

7. Dilute annealed bridge linkers to 200 ng/µl with TE buffer.

*Important! Keep all annealed bridge linkers cold on ice.*

8. Run 200 ng of each single-stranded oligos and 200 ng of the annealed bridge linkers from each of the five different annealing conditions on the same 4%-20% TBE gel. Usually, the best ratio of top oligo versus bottom oligo is the one that shows the maximal amount of annealed double-strand linker with the minimal top or bottom oligo left on the gel. For example, in the figure below, the best ratio of top versus bottom oligo is 1.5 to 1.

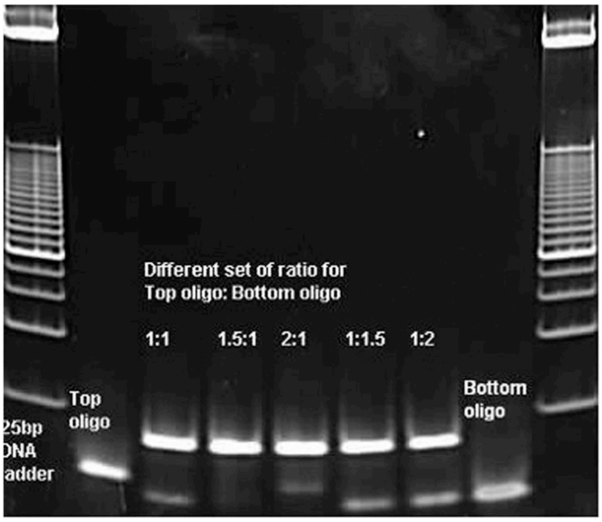


**Supplementary Figure 13.** Quality assessment for the annealing of bridge linker.

9. Perform a large-scale annealing reaction with the remaining top and bottom oligos according to the optimal ratio. For this, repeat step 5.

10. Perform Nanodrop quantification and dilute the annealed bridge linker to 2 ng/µl with TE buffer. Store at –20 °C.
