# Supplementary material for: ChIATAC is an efficient strategy for multi-omics mapping of 3D epigenomes from low-cell inputs
Source: Nat Commun. 2023 Jan 13;14:213. doi: 10.1038/s41467-023-35879-5 (PMC9839710; doi:10.1038/s41467-023-35879-5)
Supplement: Supplementary file 2 — Reporting Summary [file 41467_2023_35879_MOESM2_ESM.pdf]

nature portfolio

Corresponding author(s): Chia-Lin Wei

Last updated by author(s): Dec 25, 2022

Reporting Summary

Nature Portfolio wishes to improve the reproducibility of the work that we publish. This form provides structure for consistency and transparency in reporting. For further information on Nature Portfolio policies, see our Editorial Policies and the Editorial Policy Checklist.

Statistics

For all statistical analyses, confirm that the following items are present in the figure legend, table legend, main text, or Methods section.

n/a	Confirmed	
☐	☒	The exact sample size (*n*) for each experimental group/condition, given as a discrete number and unit of measurement
☒	☐	A statement on whether measurements were taken from distinct samples or whether the same sample was measured repeatedly
☐	☒	The statistical test(s) used AND whether they are one- or two-sided<br>*Only common tests should be described solely by name; describe more complex techniques in the Methods section.*
☒	☐	A description of all covariates tested
☒	☐	A description of any assumptions or corrections, such as tests of normality and adjustment for multiple comparisons
☐	☒	A full description of the statistical parameters including central tendency (e.g. means) or other basic estimates (e.g. regression coefficient) AND variation (e.g. standard deviation) or associated estimates of uncertainty (e.g. confidence intervals)
☐	☒	For null hypothesis testing, the test statistic (e.g. *F*, *t*, *r*) with confidence intervals, effect sizes, degrees of freedom and *P* value noted<br>*Give P values as exact values whenever suitable.*
☒	☐	For Bayesian analysis, information on the choice of priors and Markov chain Monte Carlo settings
☒	☐	For hierarchical and complex designs, identification of the appropriate level for tests and full reporting of outcomes
☐	☒	Estimates of effect sizes (e.g. Cohen's *d*, Pearson's *r*), indicating how they were calculated

Our web collection on statistics for biologists contains articles on many of the points above.

Software and code

Policy information about availability of computer code

Data collection	Illumina sequencing BCL files were converted to FASTQ with BCL2Fastq version 2.18.0.12

Data analysis	- Pair-end reads from FASTQ were processed with CPU v0.01ar2 (https://github.com/cheehongsg/CPU/releases/tag/v0.0.1a-r2) to get interaction table. This pipeline contains BWA aln and mem compiled as the mapper compiled.<br>- Peak Calling: MACS v2.2.7.1<br>- samtools v1.9<br>- bedtools v2.27.1<br>- deepTools v3.5.1<br>- Hi-C file tools: Juicer tools 1.22.01<br>- HiC reproducibility measure: HiCRep Python version https://github.com/dejunlin/hicrep<br>- HiCExplorer v3.5<br>- HOMER v4.11<br>- RNA-seq mapped with hisat v2.1.0<br>- Gene quantification: htseq-count v0.13.5<br>- R 3.5.2<br>- Differential genes: DESeq2<br>- diffTF v1.8<br>- Mustache v1.2.1<br>- Diffbind v3.4.1<br>- ChiaSigScaled v1.19.44-r2

For manuscripts utilizing custom algorithms or software that are central to the research but not yet described in published literature, software must be made available to editors and reviewers. We strongly encourage code deposition in a community repository (e.g. GitHub). See the Nature Portfolio guidelines for submitting code & software for further information.

Data

Policy information about availability of data

All manuscripts must include a data availability statement. This statement should provide the following information, where applicable:

- Accession codes, unique identifiers, or web links for publicly available datasets
- A description of any restrictions on data availability
- For clinical datasets or third party data, please ensure that the statement adheres to our policy

Data are available at GEO under accession number GSE194036. Supplemental table 1 lists all datasets generated and used in this study.

Human research participants

Policy information about studies involving human research participants and Sex and Gender in Research.

Reporting on sex and gender	N/A
Population characteristics	N/A
Recruitment	N/A
Ethics oversight	N/A

Note that full information on the approval of the study protocol must also be provided in the manuscript.

Field-specific reporting

Please select the one below that is the best fit for your research. If you are not sure, read the appropriate sections before making your selection.

☒ Life sciences ☐ Behavioural & social sciences ☐ Ecological, evolutionary & environmental sciences

For a reference copy of the document with all sections, see nature.com/documents/nr-reporting-summary-flat.pdf

Life sciences study design

All studies must disclose on these points even when the disclosure is negative.

Sample size	We used well-characterized Drosophila S2 and human GM12878 cell lines as the sample for developing a new genomic sequencing method. The cells numbers used in each experiment were sufficient and were determined using cell counter. 50,000 cells were used for S2 cells. 1,000, 10,000, 25,000, 50,000 cells were used for GM12878 cells. We then applied our new method to primary CD4+ T cells isolated from a buffy coat from a normal blood donor. 50,000 cells were used for primary human CD4+ T cells. For each sample, at least two technical replicates were included for assessing data reproducibility.
Data exclusions	No data were excluded.

Replication	Throughout the study, the reproducibility of at least two replicates was assessed by deepTools for read coverage,and HiCRep for contact maps. All replication attempts were successful.
Randomization	Randomization was not applicable, since there was no comparison between different repeated experiments.
Blinding	Data used in this study were processed and analyzed with computational tools. No blinding was required in this study. The investigators were not blinded during data collection and analysis.

Reporting for specific materials, systems and methods

We require information from authors about some types of materials, experimental systems and methods used in many studies. Here, indicate whether each material, system or method listed is relevant to your study. If you are not sure if a list item applies to your research, read the appropriate section before selecting a response.

Materials & experimental systems

n/a	Involved in the study
☐	☒ Antibodies
☐	☒ Eukaryotic cell lines
☒	☐ Palaeontology and archaeology
☒	☐ Animals and other organisms
☒	☐ Clinical data
☒	☐ Dual use research of concern

Methods

n/a	Involved in the study
☒	☐ ChIP-seq
☒	☐ Flow cytometry
☒	☐ MRI-based neuroimaging

Antibodies

Antibodies used	anti-RNAPII monoclonal antibody (8WG16, cat# 920102, Biolegend), anti-CTCF Polyclonal antibody (ABclonal, cat# A1133), anti-CD3 antibody (317353, Biolegend), and anti-CD28 antibody (302901, Biolegend) were used.
Validation	The anti-RNAPII and anti-CTCF antibodies have been validated by the ENCODE project (ENCODE accession #, ENCAB725WBH, ENCAB635SXP). The rest antibodies are commercially available,and the validations were completed by the manufacturer.

Eukaryotic cell lines

Policy information about cell lines and Sex and Gender in Research

Cell line source(s)	Drosophila Schneider 2 (S2) cells: derived from a primary culture of late stage 20-24 hours old Drosophila melanogaster embryos. Ordered from ThermoFisher Scientific (Cat# R69007).<br>Human GM12878 cells : a B-lymphoblastoid cell line produced from the blood of a female donor with northern and western European ancestry by EBV transformation. Ordered from Coriell Institute of Medical Research.
Authentication	S2 and GM12878 cells are well established cellular models for genomic analysis.<br>GM12878 cell line was purchase from Coriell Institute of Medical Research, Species of Origin Confirmed by Nucleoside Phosphorylase, Glucose-6-Phosphate Dehydrogenase, and Lactate Dehydrogenase Isoenzyme Electrophoresis. It is selected in Encode Biosample and widely applied for ChIP-seq (ENCBS830CIQ) and ChIA-PET (ENCBS502UVH).<br>Drosophila S2 cell line has been characterized by isozyme and karyotype analysis by Themo Fisher Scientific.
Mycoplasma contamination	The two cell lines used in this study were followed ENCODE and modENCODE certified cell culture protocols, and no contamination were observed. To specifically comply with the Nature policy, we tested mycoplasma contamination, and both cell lines are negative.
Commonly misidentified lines<br>(See ICLAC register)	No commonly misidentified cell lines were used in this study.
