# Supplementary material for: ChIATAC is an efficient strategy for multi-omics mapping of 3D epigenomes from low-cell inputs
Source: Nat Commun. 2023 Jan 13;14:213. doi: 10.1038/s41467-023-35879-5 (PMC9839710; doi:10.1038/s41467-023-35879-5)
Supplement: Supplementary file 3 — Description of Additional Supplementary Files [file 41467_2023_35879_MOESM3_ESM.pdf]

Title: Supplementary Data 1
Description: Output of diffTF analysis for assessing the differential chromatin accessibility and gene expression during the cell-state transition in $CD4^+$ T cells.
